# Effect of electroencephalography-guided anesthesia on neurocognitive disorders in elderly patients undergoing major non-cardiac surgery: A trial protocol The POEGEA trial (POncd Elderly GEneral Anesthesia)

**DOI:** 10.1371/journal.pone.0255852

**Published:** 2021-08-10

**Authors:** Louis Morisson, Pascal Laferrière-Langlois, François Martin Carrier, Gabrielle Pagé, Cédric Godbout, Louis-Philippe Fortier, David Ogez, Geneviève Létourneau, Stéphanie Jarry, André Denault, Annik Fortier, Marie-Claude Guertin, Olivier Verdonck, Philippe Richebé

**Affiliations:** 1 Department of Anesthesiology and Pain Medicine, Maisonneuve-Rosemont Hospital – CIUSSS de L’Est de l’Ile de Montréal, Université de Montréal, Montréal, Québec, Canada; 2 Department of Anesthesiology and Pain Medicine, Université de Montréal, Québec, Canada; 3 Department of Anesthesiology and Department of Medicine, Critical Care Division, Centre Hospitalier de l’Université de Montréal (CHUM), Université de Montréal, Montréal, Québec, Canada; 4 Research Center of the CHUM (Centre Hospitalier de l’Université de Montréal), Université de Montréal, Montréal, Québec, Canada; 5 Department of Psychology, Université de Montréal, Montréal, Québec, Canada; 6 Research Center of the CIUSSS de L’Est de l’Ile de Montréal, Université de Montréal, Montréal, Québec, Canada; 7 Department of Anesthesiology, Montréal Heart Institute, Montréal, Québec, Canada; 8 Department of Statistics, Montreal Health Innovations Coordinating Center (MHICC), Montréal, Québec, Canada; Asan Medical Center, University of Ulsan College of Medicine, REPUBLIC OF KOREA

## Abstract

**Introduction:**

The number of elderly patients undergoing major surgery is rapidly increasing. They are particularly at risk of developing postoperative neurocognitive disorders (NCD). Earlier studies suggested that processed electroencephalographic (EEG) monitors may reduce the incidence of postoperative NCD. However, none of these studies controlled for intraoperative nociception levels or personalized blood pressure targets. Their results remain unclear if the reduction in the incidence of postoperative NCD relates to avoidance of any electroencephalographic pattern suggesting excessive anesthesia depth.

**Objective:**

The objective of this trial is to investigate–in patients ≥ 70 years old undergoing major non-cardiac surgery–the effect of EEG-guided anesthesia on postoperative NCD while controlling for intraoperative nociception, personalized blood pressure targets, and using detailed information provided by the EEG monitor (including burst suppression ratio, density spectral array, and raw EEG waveform).

**Material and methods:**

This prospective, randomized, controlled trial will be conducted in a single Canadian university hospital. Patients ≥ 70 years old undergoing elective major non-cardiac surgery will be included in the trial. The administration of sevoflurane will be adjusted to maintain a BIS index value between 40 and 60, to keep a Suppression Ratio (SR) at 0%, to keep a direct EEG display without any suppression time and a spectrogram with most of the EEG wave frequency within the alpha, theta, and delta frequencies in the EEG-guided group. In the control group, sevoflurane will be administered to achieve an age-adjusted minimum alveolar concentration of [0.8–1.2]. In both groups, a nociception monitor will guide intraoperative opioid administration, individual blood pressure targets will be used, and cerebral oximetry used to tailor intraoperative hemodynamic management. The primary endpoint will be the incidence of NCD at postoperative day 1, as evaluated by the Montreal Cognitive Assessment (MoCA). Secondary endpoints will include the incidence of postoperative NCD at different time points and the evaluation of cognitive trajectories up to 90 days after surgery among EEG-guided and control groups.

**Study registration:**

NCT04825847 on ClinicalTrials.gov.

## Introduction

With the rapidly expanding ageing population, the number of elderly patients undergoing major surgery is increasing. In 2011, the proportion of people > 65 years old in the province of Québec was 1 in 6 and is expected to increase to 1 in 4 by 2031 [1]. Additionally, health-related costs are expected to exponentially increase for this sub-population [2]. This presents some major challenges as these patients are at a higher risk for perioperative and postoperative complications. Among these complications are perioperative neurocognitive disorders (NCD) which are associated with extended hospital stays, dependency on social transfer payments, decreased quality of life, and increased mortality [3].

Earlier studies have suggested that electroencephalography (EEG) guided anesthesia reduced the incidence of postoperative delirium and postoperative neurocognitive disorders (NCD) [4–6]. However, the bundle of interventions possibly used to treat monitored EEG values and characteristics in these studies does not confirm that adjusting anesthesia depth itself may reduce postoperative NCD incidence. In all studies, blood pressure management data were unavailable while managing low blood pressure instead of anesthesia depth to correct EEG-based values during surgery may be the efficacious intervention for NCD [7, 8]. Nociception levels were also never monitored, ignoring the effect intraoperative large doses of opioids or intraoperative excess of nociception might have on EEG values and postoperative NCD. Finally, results were unclear if the reduction in the incidence of perioperative NCD was related to avoidance of burst suppression–an electroencephalographic pattern suggesting excessively deep anesthesia–or treating any other EEG-based monitored value [9].

Processed EEG monitors have been used to guide the administration of hypnotic drugs to provide optimal depth of anesthesia and avoid overdoses [10]. High doses of anesthetics are directly responsible for neurotoxicity [11] and increase neuroinflammatory responses to surgical trauma [12]. Excessively deep anesthesia also causes EEG burst suppression pattern that may alter postoperative neurocognitive functions [13]. Taking into consideration the cardiovascular effects of the anesthetic agents used, EEG-guided anesthesia also may reduce episodes of hypotension or cerebral hypoxemia through hemodynamic interventions that may drive observed effects on postoperative neurocognitive outcomes.

Since 2013, and the study by Radtke et al. [6], the literature on EEG-guided anesthesia has mostly focused on postoperative delirium [14]. To our knowledge, no major trial has been published since the new nomenclature on neurocognitive disorders was proposed [15]. In addition, processed EEG monitors have become more sophisticated, allowing to display the spectral density array (DSA) extracted from the raw EEG, the burst suppression ratio, and the total burst suppression time. These details may be crucial since EEG under general anesthesia with propofol and volatile anesthetic gases vary widely with age [16] and burst suppression may be potentially associated with NCD. A recent systematic review recommended to prevent such burst suppression episodes and suggested that BIS-guided anesthesia may reduce the incidence of adverse postoperative neurocognitive outcomes such as delirium and NCD [17]. Nonetheless, the authors concluded that there remains insufficient evidence to recommend the routine use of EEG-guided anesthesia to reduce such [17].

### Objectives

The main objective of the trial is to investigate whether an EEG-guided general anesthesia administration strategy decreases the incidence of NCD at postoperative day 1 in elderly patients undergoing major non-cardiac surgery compared to standard care. The studied intervention is designed to reduce the amount of administered anesthetic at its minimal requirement to maintain an adequate anesthesia depth and minimize burst-suppression periods, while controlling hemodynamic and analgesic cointerventions. In line with recent recommendations [17], we propose to use the information provided by the BIS^™^ processed EEG monitor (Medtronic, Brampton, ON, Canada), including the BIS index value, the raw waveform, the spectrogram, and the suppression ratio to guide anesthesia in the EEG-guided group. To control cointerventions and isolate the anesthesia effect, our protocol will strictly monitor and manage nociception level, blood pressure, and cerebral oximetry with classically used algorithms (described in the method section). Secondary objectives of the trial are to evaluate, in both groups, cognitive trajectories (up to 3 months), the incidence of postoperative delirium, total intraoperative anesthetics consumption, incidences of intraoperative hypotension and cerebral hypoxemia, cumulative burst suppression duration, low BIS values during anesthesia, awareness incidence and quality of recovery. Tertiary objectives are to explore the effect of EEG-guided anesthesia compared to standard care across and within subgroups, including different categories of surgeries, duration of surgery, preoperative frailty, preoperative presence of depressive symptoms, age, and patients with preoperative neurocognitive disorders.

## Material and methods

### Ethics

Written consent will be obtained from all participants. The trial was approved by the scientific and ethical committee, Comité d’éthique de la recherche, CIUSSS de l’Est de l’Ile de Montréal, 5415, boulevard de l’Assomption on April 20^th^, 2021 (REB # 2021–2611). The trial was registered on the ClinicalTrials.gov website on March 31^st^, 2021, with registration number NCT04825847. The trial will be conducted following the Declaration of Helsinki.

### Study design

This study will be a prospective monocentric randomized controlled trial. Patients will be blinded and randomly assigned to one of the two groups (EEG-guided anesthesia with BIS *versus* standard care–SC). Processed EEG (BIS) will be monitored in both groups, but information from those monitors will only be available to clinicians for the EEG-guided group to guide hypnotic drug administration. All other aspects of anesthesia will be the same between groups, especially concerning the management of nociception, blood pressure, and cerebral oximetry.

### Randomization

Patients will be assigned to one of the two groups by a random computer-generated number using a 1:1 randomization ratio and variable random blocks of 4 and 6. Randomization will occur after consent for trial participation is obtained and a preoperative visit is conducted. An envelope containing the trial group will be opened after inclusion. Randomization will be communicated to the anesthesia team only just before the patient is sent to the operating room.

### Blinding

Patients and research assistants performing cognitive assessments (pre- and post-operatively) will be blinded to the trial group. The anesthesiologist in charge of the patient during the anesthesia and surgery will be aware of the patient trial group but will not take part in the postoperative outcome ascertainment.

### Participants

All consecutive patients ≥ 70 years old who are scheduled for prespecified major surgeries and meeting our inclusion criteria will be approached and included if they consent. We aim to include a total of 314 patients (157 per group) (see justification below).

#### Inclusion criteria

- Patients ≥ 70 years of age,
- Major gynecologic, abdominal, urologic, thoracic, or orthopedic surgery via laparoscopy or laparotomy under general anesthesia (with or without concomitant use of regional or neuraxial anesthesia),
- Expected anesthesia time of > 60 min,
- Seen for assessment by internal medicine and/or anesthesiology at the preoperative clinic (CIEPC).

#### Exclusion criteria

- Known diagnosis of dementia or other neurological, psychiatric, developmental, or medical condition that resulted in documented severe cognitive impairment,
- Emergency surgery,
- Significant auditory or visual impairment that precludes participation in cognitive testing,
- Known allergy, intolerance, other medical condition that precludes the use of prescribed general anesthesia protocol for this trial,
- Inability to communicate in French or English.

### Anesthesia protocol

#### Monitoring

Routine monitoring of patients will be the same for both groups and will include 5-lead ECG, non-invasive blood pressure, pulse oximetry, and a temperature probe. We will record all data using the Dräger Perseus A500 (Dräger Medical, Lübeck, Germany) monitor and send all anesthesia-related data electronically to a research computer for anonymized storage and further analysis.

We will place the NOL index finger probe (PMD-200™ device, Medasense Biometrics Ltd, Ramat Gan, Israel) and the bilateral cerebral regional oximetry rSO_2_ (Invos™, Medtronic, Brampton, ON, Canada) (both are available to the anesthesiologist for both groups throughout the entire anesthesia). A bilateral BIS^™^ EEG sensor (Medtronic, Brampton, ON, Canada) will be placed on the forehead for all patients. For the SC group, only the electrode status display will be available; the rest of the data will be hidden by a dark screen and unavailable to the anesthesiologist during the anesthesia.

#### Anesthesia

All patients will undergo general anesthesia using the following procedures: induction with intravenous (IV) slow boluses of lidocaine to numb the vein, propofol 1.5mg.kg^-1^, remifentanil 1μg.kg^-1^ over 30 sec, rocuronium 0.8 mg.kg^-1^. We will proceed to orotracheal intubation when a response to neuromuscular stimulation of the adductor pollicis has reached 5% of the original T4 amplitude. The maintenance of anesthesia will be done using sevoflurane to reach [0.8–1.2] minimal alveolar concentration (MAC; MAC adjusted to the patient’s age) in the control group, and to achieve a BIS index of [40–60] in the EEG-guided intervention group (see below).

We will administer intravenous boluses of rocuronium as needed for the response to TOF stimulation to stay below 2/4. Benzodiazepines or ketamine will not be used, and patients will receive a standard prophylaxis against postoperative nausea and vomiting (avoiding IV haloperidol). Remifentanil and phenylephrine IV infusions will be discontinued when the skin is closed. At the same time, muscle relaxation will be antagonized using IV neostigmine 0.04 mg.kg-1 and glycopyrrolate 0.01 mg.kg-1, and a bolus of IV ondansetron 4 mg and hydromorphone 0.006 μg.kg-1 will be administered. Sevoflurane will be discontinued when the wound dressing is completed, and patients will be extubated in the operating room and transferred to the post-anesthesia care unit (PACU).

Hydromorphone PCA (or SC hydromorphone if the patient is not a candidate for PCA) for postoperative pain scores < 4/10, or PCEA (if an epidural is placed) will be started in PACU. All patients will be managed according to the ERAS (Enhanced Recovery after Surgery) principles as per routine care in our center. All anesthesia-related side effects, quality of analgesia, and rehabilitation will be evaluated for 48 h (see below). As stated before, same cointerventions regarding opioid use and hemodynamic management will be applied to either group.

### Nociception management

The remifentanil infusion will start at 0.02 μg.kg^-1^.min^-1^ and increase by 0.02 μg.kg^-1^.min^-1^ according to the NOL index. If the NOL index is > 25 for more than 1 min, we will administer one bolus of 0.3 μg.kg^-1^. The maximum remifentanil rate allowed intraoperatively will be 0.3 μg.kg^-1^.min^-1^. This rate will be decreased by step of 0.02 μg.kg^-1^.min^-1^ if the NOL index is < 5 for more than 3 min (for a minimum rate of 0.02 μg.kg^-1^.min^-1^).

#### Hemodynamic management

Baseline mean arterial blood pressure (MAP) will be defined as the average of three consecutive values taken 1 min apart and determined before the induction of general anesthesia. Intravenous (IV) infusion of phenylephrine will start at 0.2 μg.kg^-1^.min^-1^ and adjusted to maintain +/- 20% of the baseline values of the pre-anesthesia MAP. Intraoperative hypovolemia will be screened using repeated measures of pulse pressure variation and intravenous fluid boluses will be allowed and administered at the discretion of the anesthesiologist in charge. Perioperative blood management will be done according to guidelines [18].

Hemodynamic management algorithms will be provided to clinicians so that cerebral oximetry values will be the same in both groups.

#### Intervention group

In the EEG-guided group, the information provided by the BIS^™^ monitor will guide the volatile anesthetic administration. Sevoflurane end-tidal anesthetic concentration (ETAC) will be adjusted to maintain a BIS value between 40 and 60, a Suppression Ratio (SR; % of time with suppressed brain electrical activity) at 0%, a direct EEG display without any suppression time and a spectrogram (DSA or density spectral array) with most of the EEG wave frequency within the Alpha (8-12Hz), Theta (4-8Hz) and Delta (0.5-4Hz) frequencies.

#### Control group

In the non-EEG-guided group, the MAC of sevoflurane will be used to guide the intraoperative anesthesia delivery and kept between 0.8 and 1.2 (age-adjusted). The BIS index will be recorded but it will not be available to the anesthesiologist intraoperatively.

### Outcomes

#### Primary outcome

- Incidence of major NCD at postoperative day 1.

#### Secondary outcomes

- The incidence of major NCD at postoperative day 2, 7, 15, 30 and 90
- The evolution over time in cognitive assessment scores defining cognitive trajectories,
- The incidence of postoperative delirium during hospital stay,
- The total intraoperative consumption of volatile anesthetics, opioids, and vasopressors over surgery time,
- The incidence of intraoperative hypotension,
- The incidence of cerebral hypoxemia,
- The durations of cumulative burst suppression and low BIS values during anesthesia,
- The incidence of awareness,
- The difference between the quality of recovery scores between groups.

#### Tertiary outcomes

- Incidence of major NCD at postoperative day 1, 2, 7, 15, 30 and 90 among the following subgroups of patients: different surgical types and duration, patients with a preoperative neurocognitive disorder (defined as a preoperative MoCA score < 26 [19]), frail patients (defined as a CFS ≥ 5 [20]), depressive patients (defined as a PHQ-9 score ≥ 10 [21]), and patients > 80 years old.

### Outcome measurement

#### Cognitive assessments

*The Montreal cognitive assessment tool*. We will perform preoperative assessment the morning of the scheduled surgery and postoperative assessments on days 1, 2, 7, 15, 30 and 90. All cognitive assessments will use the Montreal Cognitive Assessment (MoCA) tool. The MoCA takes roughly 10 min to administer and covers eight cognitive domains. It was originally designed as a screening tool to detect mild cognitive impairment (MCI) [19] but it is also used in clinical and research assessment of a variety of neurological and cognitive pathologies. A score below 26 is considered positive for cognitive impairment. It has three versions, allowing for retesting with a low risk of learning between assessments. The telephone version of the MoCA (T-MoCA) is scored on a scale of 0 to 22 and excludes the visuospatial/executive and naming component. The T-MoCA has proved to be a reliable method to assess MCI after stroke and has been used to explore cognitive functions after major elective surgery [22].

In our previous trial (data not published yet), we encountered difficulties administering MoCA to patients who had left the hospital. So, for our proposed trial, we chose to use MoCA for in-hospital cognitive assessments and the T-MoCA once patients returned home. The MoCA scores will be used for the primary endpoint assessment–pre-anesthesia *versus* 24 h after surgery–and T-MoCA scores will be used to establish the cognitive trajectories up to three months after general anesthesia for major surgery in this elderly population.

*Verbal fluency*. Testing verbal fluency requires a participant to generate as many words as possible within set times and parameters, such as, phonemic verbal fluency: *e*.*g*., words that start with the letter f, and categorical verbal fluency: *e*.*g*., names of animals. These verbal tasks evaluate lexical access but also assess sustained attention and memory. These tests can be completed in person or over the phone and have proven reliable means of administering the test in both French and English [23–26] and are excellent tools to assess a variety of cognitive impairments across many disorders (MCI, Alzheimer Dementia, traumatic brain injury, Parkinson’s disease).

*Definition of neurocognitive disorders*. We will calculate Z-scores for individual tests (Verbal fluency, MoCA, T-MoCA) at each testing time point using the mean and standard deviation (SD) of baseline tests of all patients:

$$Z_{test}=\frac{x_{test}-\mu_{test\;baseline}}{\sigma_{test\;baseline}}$$

where *x* corresponds to individual test value, *μ* and *σ* correspond to test mean, and SD of all patients at baseline respectively.

We will use MoCA Z-scores (or T-MoCA depending on the testing time point) to define NCD. Neurocognitive disorders will be classified as mild or major (Table 1) [15, 27]:

- **No NCD**: a decrease in Z-score < 1 SD,
- **Mild:** a decrease in Z-score ≥ 1 SD,
- **Major:** a decrease in Z-score ≥ 1.96 SD.

**Table 1:** Primary outcome definition.

Z-score decrease	Postoperative days 1,2,7,15, 30 and 90
**< 1 SD**	No NCD
**≥ 1 SD and < 1.96 SD**	Mild NCD
**≥ 1.96 SD**	Major NCD

SD: standard deviation. NCD: neurocognitive disorder

#### Other assessments

*Preoperative assessment of depression and frailty*. We will pre-screen for symptoms of severe depression and extreme fragility at preoperative enrollment to ensure cognitive impairment is not related to these two conditions. We will use the Patient Health Questionnaire 9 (PHQ-9) to detect the severity of depression [28] and the Clinical Frailty Scale 2.0 [29] to assess frailty. Both screening tools are easy to use for clinicians, are validated, and available in both English and French [30, 31].

*Delirium assessment*. We will administer the Confusion Assessment Method (CAM) on postoperative days 1 and 2. The CAM is a validated assessment tool for the diagnosis of delirium [32] and is based on criteria from the Diagnostic and Statistical Manual of Mental Disorders (DSM). The cognitive testing on postoperative days 1 and 2 will consist of basic orientation questions and a sustained attention task (stating the months of the year backwards). If the CAM yields a positive diagnosis, we will then administer the CAM-S which assesses the severity of delirium. In the case of persistent mechanical ventilation after surgery in the intensive care unit (ICU), we will administer the ICU version of the CAM (CAM-ICU) [33].

*Postoperative assessment of quality of life*. We will perform the Quality of Recovery-15 (QoR-15) interview at postoperative days 30 and 90 to explore quality of life after anesthesia and surgery and to assess differences between groups. This is a validated shorter version of the QoR-40 with only 15 items [34].

#### Research timeline

*Baseline visit*. All eligible patients will be evaluated and recruited at the preoperative clinic. Patients will be screened, and the consent form provided at this visit. On the day of surgery (D0), after confirming consent, patients will undergo the inclusion visit where a cognitive assessment will be performed using the Montreal Cognitive Assessment (MoCA) [19] and verbal fluency tests conducted by a trained research team. Patients will also be screened for depression and frailty. The patient will then be randomized into the EEG-guided or SC group.

**Fig 1:**
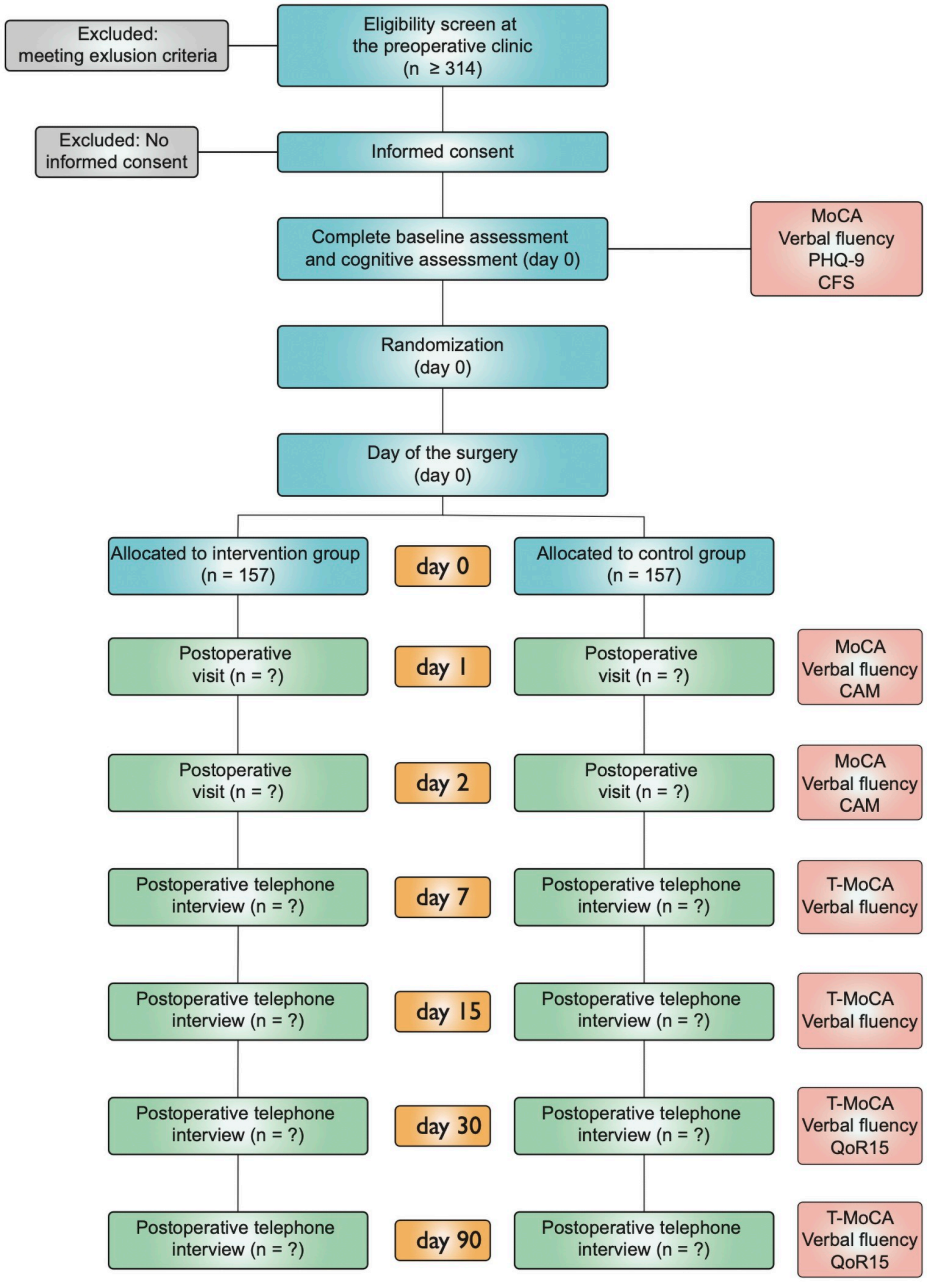
SPIRIT schedule of enrollment, interventions, and assessments. MoCA: Montréal Cognitive assessment, PHQ-9: Patient Health Questionnaire 9, CAM: Confusion Assessment Method, T-MoCA: Telephone MoCA, QoR15: Quality of Recovery 15.

*Outcome ascertainment timeline*. Visits will be performed on postoperative days 1 and 2 (D1 and D2). Cognitive assessments will again be conducted using MoCA and verbal fluency tests. Patients will also be screened for postoperative delirium. Once patients return home, phone interviews will be done at D7, D15, D30 and D90. Cognitive assessments will be performed using the telephone version of the MoCA (T-MoCA) and the verbal fluency tests. The quality of recovery will be evaluated at D30 and D90 using the QoR-15 questionnaire [34]. The T-MoCA as well as the verbal fluency will be used to establish cognitive trajectories of the two groups. The three versions of the T-MoCA will be alternately and randomly used to prevent any learning effect. The estimated time for each visit is approximately 45 min. All the tests are provided in the references and detailed below. The SPIRIT (Standard Protocol Items: Recommendations for Interventional Trials) schedule of enrollment, interventions, and assessments is presented in Fig 1.

### Safety

The research personnel and hospital staff will make every effort to ensure the safety of the participants. We expect no adverse events to result from this research.

#### Anesthesia management protocol

The anesthetic management in the proposed trial represents a standard and conventional approach to general anesthesia (see “Risks” section below). There is no additional anesthesia-related patient risk arising from participation in this trial.

#### Cognitive assessment

During recruitment, we will explain to all patients that participation in the trial is voluntary, non-binding, and that cognitive assessment performance has no bearing on medical treatment. The cognitive tests used in the proposed trial will require the participant to verbally interact with the examiner and, for a few tasks, write words or draw images–the risk of physical harm from testing is minimal or non-existing. We will schedule (to the best of our ability) postoperative testing when patients are awake, comfortable, and not slated for any treatments (dressing changes, physiotherapy, etc.).

#### Medical monitoring

The principal investigator (PI) will regularly review all data (weekly or bi-weekly), including completeness of trial data, enrolment, protocol deviations, dropouts, adverse events, and create an annual report of these events. A summary of the investigation will be submitted to the approving Internal Review Board (IRB).

#### Definition of adverse events

There are no anticipated adverse events for this research. If adverse events occur, the PI will report it to the approving IRB. Adverse events will be graded as Mild, Moderate, or Serious, and Related, Possibly Related, or Not Related to trial procedures.

*Adverse event*. Any unfavourable and unintended sign, symptom, or disease temporarily associated with the use of medical treatment or procedure.

*Serious adverse event*. Any adverse event that results in any of the following outcomes:

- Outpatient hospitalization or prolongation of existing hospitalization,
- Persistent or significant disability/incapacity,
- Death.

We will monitor all adverse events that are at least possibly related to the intervention.

#### Risk

There is very little risk involved with participation in this study and the protocol is not expected to increase the duration of anesthesia. The anesthetic care provided will be routine, will use standard intraoperative monitoring, and only use common analgesics, such as intravenous remifentanil.

### Statistical plan

#### Sample size

In a previous study at HMR / CEMTL (manuscript in preparation), we evaluated (as an exploratory outcome) NCD in a population (mean age of 65) undergoing major colorectal surgery under general anesthesia. We measured a cumulative incidence of major NCD (using pre- *versus* 24 h post-surgery MMSE testing; Z-score ≥ 1.96 SD in MMSE evaluations pre- and postoperatively) at postoperative day 1 of 3.8% in the monitored group (EEG-guided anesthesia with BIS index) versus 15.4% in the control group (standard of care group, BIS placed but not used to guide intraoperative administration of the halogenous gas). Similar results were also observed by Hou R. et al. in 2018 [35].

Based on a 2-sided α <0.05 and 80% power, we calculated that 282 patients were required to detect (as the primary endpoint of the proposed study) a minimal clinically significant difference of 10% of NCD incidence in the EEG-guided group compared to the control group (expected decrease from 15% to 5% or less). The sample size was inflated to 314 (157 in each group) to account for a 10% withdrawal or loss of follow-up.

#### Statistical methods

*General considerations*. Descriptive statistics will be presented by groups using mean (SD) or median (Q1, Q3) for continuous parameters (according to the skewness of the distribution of each parameter) and proportions for categorical parameters. According to the nature of the analyzed outcomes, risk differences between proportions or mean (or median) differences with 95% confidence intervals (CIs) will be presented. The alpha value will be set a 0.05 and all statistical analysis will be performed using SAS, SPSS, or R software.

*Primary analysis*. Our primary analysis will be an intention-to-treat analysis on the risk difference of NCD between groups at postoperative day 1. Incidence of NCD will be presented using frequency and proportion (%) broken down by randomized groups. The incidence of NCD will be compared between the two groups using a chi-square test. A risk difference will be reported with a score (Wilson) 95% confidence interval that has an unbiased coverage in rare event situations.

In the case we observe relevant quantitative imbalances in prognostic factors between the treatment groups, we will conduct an adjusted propensity score-based analysis of the primary outcome. We will estimate a standardized marginal average treatment effect (risk difference) model adjusted from 10 strata based on an estimated propensity score from unbalanced NCD risk factors (age, sex, preoperative MoCA score, CFS, PHQ-9, and education level). The marginal risk difference will be reported with 95% confidence intervals, estimated by non-parametric bootstrap (1000 replications).

*Secondary analyses*. We will present results and group comparisons (EEG use or SC) for the secondary dichotomous outcomes using the same approach described in the primary analysis. For the continuous endpoints, group comparisons will be completed using a t-test or a Mann-Whitney-Wilcoxon test, according to the distribution of each variable, and a chi-square test will be used for proportion. Risk differences or mean differences with 95% confidence intervals will be reported for all outcomes.

*Trajectories analyses*. Z-scores of T-MoCA test (baseline, D1, D2, D7, D15, D30 and D90) will be entered in a group-based trajectory model (GBTM) to derive trajectories of cognitive function. GBTM allows for the identification of patient subgroups sharing similar cognitive patterns over time [36, 37]. Different models will be tested that vary based on the number of trajectories (models that contain between 1 and 8 trajectories will be tested) and inclusion of linear and quadratic terms. The selection of the final model is based on Bayesian Information Criteria (BIC), parsimony, and ≥ 5% of patients in each class. Once the final model is selected, outcome variables represent probabilities of belonging to each of the trajectories. For each of the relevant baseline variables, the GBTM obtained will be rerun with the inclusion of age, sex, education, and randomization group as covariates. For each model, an odds ratio will examine the contribution of the variable of interest to the classification of patients in each trajectory.

*Subgroups analyses*. Subgroup analyses will be conducted in the following subgroups: different surgical categories and duration, patients with preoperative neurocognitive disorders (defined as a preoperative MoCA score < 26), frail patients (defined as a CFS ≥ 5), depressive patients (defined as a PHQ-9 score ≥ 10) and patients > 80 years old. Stratified treatment effects (risk difference) will be reported for our primary outcome within each subgroup with 95% confidence intervals as well as the result of an interaction test between subgroup characteristics and allocated treatment.

### Data management protocol

We will collect all electronic data from the medical monitoring equipment, including BIS^™^ and PMD-200^™^. Monitor times will be synchronized before any data is collected. We will note, on a separate Case Report Form (CRF) and in the integrated system of PMD-200^™^, all anesthesia- and surgery-related events. At the end of anesthesia, and once the patient is extubated, we will export all electronic data. Data will be anonymized and stored in a dedicated research computer at the Department of Anesthesiology and Pain Medicine of HMR-CEMTL.

We will assign data unique study codes (P1, P2, and so on up until PX) that link to the subject’s identity. This link will be kept separately to avoid patient identification in the event of theft or loss of data. Protected health information will not be re-used by our institution or disclosed to a third party, (except as required by law), for authorized oversight of the research, or as permitted by a written authorization signed by the research subject. Data will be stored on a password-protected laptop which has been assigned to the PI who will maintain primary responsibility for this computer and the data. Paper copies (consent forms) of patient information will be stored in a locked file cabinet and office in the Department of Anesthesiology and Pain Medicine. Only the PI will have access to all the data during the study.

Every person involved in this study will receive appropriate training, abide by confidentiality guidelines to protect the subject’s privacy, and strictly follow rules and guidelines as outlined in the Health Information Protection Act (Canada).

### Study monitoring and timeline

Every month the PI will review the recruitment and data collection. The trial will start in **September** 2021 and run for two years. Three months of patient follow-up will be added at the end of inclusions. Data analysis, manuscript writing, and submission for publication will follow at the end of 2023.

## Discussion

Several studies investigated the effect of EEG-guided anesthesia on postoperative NCD. A recent meta-analysis concluded that there remains insufficient evidence to recommend the routine use of EEG-guided anesthesia to reduce postoperative NCD [17]. The largest study performed by Radtke et al. did not find any benefit in monitoring EEG during anesthesia on postoperative NCD [6]. One significant limitation of this previous study is that the mean BIS values were similar in the BIS-guided and control groups, raising questions about anesthesiologists’ compliance with the study protocol.

One strength of our study design is that we have a very well-trained team in EEG-guided anesthesia since BIS^™^ monitors have been available in each operating room for many years, which might ease the compliance of anesthesiologists to the present trial protocol. Also, the monocentric design of our study reduces heterogeneity between different anesthesia teams and centers’ practices.

Another strength of our study is that we will use the detailed information provided by the EEG monitor, including burst suppression ratio, density spectral array, raw EEG waveform, and total burst suppression time to guide anesthesia and not simply the processed EEG BIS index value, as previous studies relied on. This is a major difference between our proposed study and earlier studies.

Finally, none of these previous studies controlled for both intraoperative analgesic administration and personalized mean blood pressure objectives. In our proposed study, remifentanil infusion will be guided in both groups on a nociception index, the NOL index. The NOL index is a tested and validated measure that shows a high correlation between pain measurements and doses of intraoperative opioids and is commonly used in our center at Maisonneuve-Rosemont Hospital / CEMTL [38, 39]. Moreover, mean blood pressure targets will be personalized, and cerebral oximetry monitoring used to ensure comparability of the two groups regarding intraoperative hemodynamic management. We believe that this strategy will allow us to better explore and isolate the effect of EEG-guided anesthesia depth on postoperative NCD.

Our study also has limitations: First, it is monocentric, and the results may not be generalizable to other centers. Nevertheless, if the study is clinically significant, it will be used to develop a multicenter project to demonstrate external validity. Second, we choose to use the MoCA test to evaluate neurocognitive functions and not an array of neuropsychological tests for practical reasons. Third, we will perform the baseline cognitive function evaluation the day of the surgery also for practical reasons. Patient level of anxiety could impair patient performance on these tests and thus decrease our ability to identify postoperative NCD. Fourth, we choose postoperative day 1 to assess the primary outcome, which is inconsistent with other published studies., but may ensure feasibility. Moreover, the incidence of neurocognitive disorder at postoperative day 1 in a previous study allowed us to calculate the sample size in the POEGEA protocol. Cognitive functions will also be evaluated at postoperative days 2, 7, 15, 30 and 90 which will be used for sample size estimation in a future multicentric RCT. Fifth, the results of the study will depend on anesthesiologists’ compliance with our protocol, but we believe that our team is strongly trained in EEG-guided administration of anesthetics and on other monitoring equipment used in the study. Finally, the design of the study requires that the anesthesiologist in charge will not be blind to the treatment group, which may introduce a “cointervention bias”. However, we controlled important cointerventions to limit such biases.

## Conclusion

The rapidly expanding ageing population makes patients undergoing major surgery at risk of neurocognitive complications. We believe that a personalized intraoperative care may contribute to reduce these complications. The POEGEA trial will investigate precisely the impact of EEG-guided anesthesia on postoperative NCD while controlling for intraoperative nociception and personalized blood pressure targets. The results of this trial may provide sufficient evidence of the routine use of processed EEG monitors for the prevention of postoperative NCD in elderly patients undergoing major non-cardiac surgery.

## Supporting information

S1 File SPIRIT checklist. (DOC)

S2 File Study protocol approved by the local IRB. (DOCX)

S3 File IRB approval letter. (PDF)
