# Supplementary material for: Effect of electroencephalography-guided anesthesia on neurocognitive disorders in elderly patients undergoing major non-cardiac surgery: A trial protocol The POEGEA trial (POncd Elderly GEneral Anesthesia)
Source: PLoS One. 2021 Aug 10;16(8):e0255852. doi: 10.1371/journal.pone.0255852 (PMC8354438; doi:10.1371/journal.pone.0255852)
Supplement: S2 File — (DOCX) [file pone.0255852.s002.docx]

Study Protocol

Effect of electroencephalography-guided anesthesia on neurocognitive disorders in elderly patients undergoing major non-cardiac surgery: a randomized clinical trial

The POEGEA study (POncd Elderly GEneral Anesthesia)

Version: 7.0

April 05^th^, 2021

ClincialTrials.gov ID: NCT04825847

**Title**

Effect of electroencephalography-guided anesthesia on neurocognitive disorders in elderly patients undergoing major non-cardiac surgery: a randomized clinical trial.

Version 7.0, April 5^th^, 2021

Principal investigator:

Philippe Richebé, MD, PhD, Full Professor with tenure, Anesthesiologist,

Research Chair, Department of Anesthesiology and Pain Medicine at University of Montréal

Maisonneuve-Rosemont Hospital – CIUSSS de l’Est de l’Ile de Montréal

(HMR- CEMTL)

5415, boulevard de l'Assomption, Montréal, Québec

H1T 2M4, Canada

+1-514-743-6558

[philippe.richebe@umontreal.ca](mailto:philipperichebe@live.com)

**Co-Investigators:**

***Will collaborate and be involved in patients’ inclusion/follow-up at HMR-CEMTL:***

Louis Morisson, MD, MSc, Anesthesia Fellow, University of Montréal

Cédric Godbout, MD, Assistant Professor, Anesthesiologist, University of Montréal

Pascal Laferrière-Langlois, MD, MSc student, Assistant Professor, Anesthesiologist, University of Montréal

Louis-Philippe Fortier, MD, MSc, Anesthesiologist, Associate Professor, University of Montréal

Olivier Verdonck, MD, MSc, Chief of the Department of Anesthesiology, Maisonneuve-Rosemont Hospital, Assistant Professor, Anesthesiologist, University of Montréal

David Ogez, PhD, Psychologist, Assistant Professor, Assistant Professor, University of Montréal

Jibba Amraoui, MD, Anesthesia Fellow, University of Montréal

Nadia Godin, Research Coordinator and RN

Moulay Idrissi, BEng, MSc, Research Assistant

**Collaborators and external consultants with specific expertise in the field:**

***Will collaborate, but not involved in patients’ inclusion/follow-up at HMR-CEMTL***

Han-Ting Wang, MD, MSc, Internal Medicine and Intensivist, Assistant Professor, University of Montréal (expert in patients’ frailty)

François-Martin Carrier, MD, MSc, Associate Professor, Anesthesiologist and Intensivist, University of Montréal (expert in methodology of RCT)

Gabrielle Pagé, PhD, Psychologist, Assistant Professor, University of Montréal (expert in biostatistics, methodology, perioperative trajectories)

Geneviève Létourneau, MD, MSc, Psychiatrist, Associate Professor, University of Montréal (expert in cognitive functions in the elderly population)

André Denault, MD, PhD, Full Professor with tenure, Anesthesiologist and Intensivist, University of Montréal (expert in cerebral monitoring)

Annik Fortier, MSc, Department of Statistics MHICC, Senior Biostatistician, University of Montréal

Marie-Claude Guertin, PhD, Department of Statistics MHICC, Chief Biostatistician, University of Montréal

**TABLE OF CONTENTS**

[A . Introduction 13](#_Toc68178351)

[A.1 Summary 13](#_Toc68178352)

[A.2 Hypothesis 14](#_Toc68178353)

[B . Background 15](#_Toc68178354)

[B.1 Prior Literature 15](#_Toc68178355)

[B.1.1 Definition 15](#_Toc68178356)

[B.1.2 Epidemiology 15](#_Toc68178357)

[B.1.3 Risk Factors and Anesthetic Management 16](#_Toc68178358)

[B.1.4 Anesthesia Depth 17](#_Toc68178359)

[B.2 Study Rationale 18](#_Toc68178360)

[C . Study Objectives 20](#_Toc68178361)

[C.1 Primary Objective 20](#_Toc68178362)

[C.2 Secondary Objectives 20](#_Toc68178363)

[C.3 Tertiary objectives 20](#_Toc68178364)

[D . Methods 21](#_Toc68178365)

[D.1 Study design 21](#_Toc68178366)

[D.2 Subject population 21](#_Toc68178367)

[D.2.1 Inclusion criteria 21](#_Toc68178368)

[D.2.2 Exclusion criteria 22](#_Toc68178369)

[D.2.3 Recruitment 22](#_Toc68178370)

[D.3 Research plan and timeline 23](#_Toc68178371)

[D.4 Anesthesia Protocol 24](#_Toc68178372)<

[D.4.1 General 24](#_Toc68178373)

[D.4.2 Monitoring 26](#_Toc68178374)

[D.4.3 Intervention 26](#_Toc68178375)

[D.5 Randomization and blinding 27](#_Toc68178376)

[D.6 Measurements 27](#_Toc68178377)

[D.6.1 Cognitive Assessments 27](#_Toc68178378)

[D.6.1.1 The Montreal Cognitive Assessment tool 27](#_Toc68178379)

[D.6.1.2 Verbal Fluency 28](#_Toc68178380)

[D.6.1.3 Z Scores and Definition of Neurocognitive Disorders (NCD) 28](#_Toc68178381)

[D.6.2 Other Assessments 29](#_Toc68178382)

[D.6.2.1 Preoperative Assessment of Depression and Frailty 29](#_Toc68178383)

[D.6.2.2 Delirium Assessment 29](#_Toc68178384)

[D.6.2.3 Postoperative Assessment of Quality of Life 30](#_Toc68178385)

[D.6.3 Endpoints 30](#_Toc68178386)

[D.6.3.1 Primary Endpoint 30](#_Toc68178387)

[D.6.3.2 Secondary Endpoints 30](#_Toc68178388)

[D.6.3.3 Tertiary endpoints 30](#_Toc68178389)

[E . Subject Visits 31](#_Toc68178390)

[E.1 Data Collection Procedures 31](#_Toc68178391)

[E.2 Safety and Adverse Events 31](#_Toc68178392)

[E.2.1 Anesthesia Management Protocol 31](#_Toc68178393)

[E.2.2 Cognitive Assessment 32](#_Toc68178394)

[E.2.3 Medical Monitoring 32](#_Toc68178395)

[E.2.4 Definition of Adverse Events 32](#_Toc68178396)

[F . Proper Handling of Subjects 33](#_Toc68178397)

[F.1 Ethical Considerations 33](#_Toc68178398)

[F.2 Subject Withdrawal 33](#_Toc68178399)

[F.2.1 Early Withdrawal of Subjects 33](#_Toc68178400)

[F.2.2 When and How to Withdraw Subjects 33](#_Toc68178401)

[F.2.3 Data Collection and Follow-up for Withdrawn Subjects 33](#_Toc68178402)

[F.3 Risks 34](#_Toc68178403)

[G . Statistical Plan 34](#_Toc68178404)

[G.1 Sample Size 34](#_Toc68178405)

[G.2 Statistical Methods 35](#_Toc68178406)

[G.2.1 General Considerations 35](#_Toc68178407)

[G.2.2 Primary Analysis 35](#_Toc68178408)

[G.2.3 Secondary Analysis 36](#_Toc68178409)

[G.2.4 Trajectories Analysis 36](#_Toc68178410)

[G.2.5 Subgroups and sensitivity analyses 36](#_Toc68178411)

[H . Data Handling and Record Keeping 37](#_Toc68178412)

[H.1 Confidentiality and Security 37](#_Toc68178413)

[H.2 Electronic Data 37](#_Toc68178414)

[H.3 Hard Data 37](#_Toc68178415)

[H.4 Coding Data 38](#_Toc68178416)

[H.5 Accessibility 38](#_Toc68178417)

[H.6 Training 38](#_Toc68178418)

[H.7 Linked Data 38](#_Toc68178419)

[I . Study Monitoring, Auditing and Inspecting 38](#_Toc68178420)

[I.1 Study Monitoring Plan 38](#_Toc68178421)

[I.2 Auditing and Inspecting 38](#_Toc68178422)

[J . Study administration 39](#_Toc68178423)

[J.1 Organization and Recruiting Site, IRB 39](#_Toc68178424)

[J.2 Study Timetable 39](#_Toc68178425)

[K . Publication Plan 39](#_Toc68178426)

[L . Budget 39](#_Toc68178427)

[M . References 40](#_Toc68178428)

Protocol summary

Title

Effect of electroencephalography-guided anesthesia on neurocognitive disorders in elderly patients undergoing major non-cardiac surgery: a randomized clinical trial.

Background

As the population ages, the number of elderly patients undergoing major surgery is increasing. Perioperative neurocognitive disorders (NCD) are common complications and can be associated with increased mortality, longer hospital stays, increased dependency and decreased quality of life^1^. Earlier studies have reported that electroencephalography (EEG) guided anesthesia reduced the incidence of both postoperative delirium and postoperative neurocognitive disorders (NCD)^2-4^. But these studies did not control for factors such as blood pressure and nociception level, while they may represent confounding factors for the real etiology of perioperative NCD. The recent ENGAGES trial failed to demonstrate any benefit of EEG-guided anesthesia as they aimed at avoiding intraoperative burst suppression, but they only focused on postoperative delirium ignoring other NCD^5^.

We designed this prospective randomized controlled trial (RCT) to explore the impact of EEG-guided anesthesia versus standard of care (SOC) on NCD evaluated at postoperative day 1 on the Montreal Cognitive assessment (MoCA) in elderly patients undergoing major non-cardiac surgery with tight control of possible confounding factors such as intraoperative blood pressure, cerebral oximetry and nociception level.

Specific Objectives

**The main objective** of the study is to investigate the effect of EEG-guided anesthesia aiming at reducing anesthetic administration and minimizing burst suppression on the EEG during general anesthesia on **the incidence of NCD** at postoperative day 1 in elderly patients (> 70 years old) undergoing major (expected duration > 1h) non-cardiac surgery compared to standard of care.

Secondary objectives are to investigate the effect of EEG-guided anesthesia compared to standard of care on:

- neurocognitive disorders at postoperative day 2, 7, 15, 30 and 90,
- perioperative cognitive trajectories,
- postoperative delirium,
- intraoperative consumption of volatile anesthetics, opioids and vasopressors,
- intraoperative hypotension (number of interventions to treat hypotensive events),
- cerebral hypoxemia (assessed by cerebral oximetry),
- cumulative burst suppression duration and cumulative low processed EEG values duration during anesthesia,
- awareness
- postoperative surgical and quality of life / recovery outcomes.

Tertiary objectives are to:

- explore the effect of EEG-guided anesthesia compared to standard of care across and within subgroups including different categories of surgical, duration of surgery, preoperative frailty, preoperative presence of depressive symptoms, age and patients with preoperative neurocognitive disorder.

Methods

Participants

314 patients 70 years of age or older scheduled for elective major gynecologic, abdominal, urologic, thoracic or orthopedic surgeries via laparoscopy or laparotomy under general anesthesia and an anesthesia time of more than 60 minutes at the institution HMR-CEMTL, who are seen at the preoperative clinic (CIEPC) by internal medicine and/or anesthesiology.

*Exclusion criteria*: patients with known diagnosis of dementia or other neurological, psychiatric, developmental or medical condition that resulted in severe documented cognitive impairment, emergency surgery, significant auditory or visual impairment that precludes participation in cognitive testing, known allergy or intolerance or other medical condition that precludes the use of prescribed general anesthesia protocol for this study, inability to communicate in French or English will not be included in the present study.

Anesthesia Protocol

All patients will undergo general anesthesia with the following: induction with IV slow boluses of lidocaine to numb the vein, propofol 1.5mg.kg^-1^, remifentanil 1µg.kg^-1^, rocuronium 0.8mg.kg^-1^. The maintenance of anesthesia will be based on sevoflurane to reach [0.8-1.2] minimal alveolar concentration (MAC adjusted to age) in the control group, and to achieve a *Bispectral index (BIS)* of [40-60] in the EEG-guided group (see below). The NOL index (PMD200™ device, Medasense Biometrics Ltd, Ramat Gan, Israel) and the bilateral cerebral regional oximetry (rSO_2_) (Invos™, Medtronic, Canada) will be placed and available for both groups throughout the entire anesthesia. Remifentanil infusion will be set between 0.02 and 0.3 µg.kg^-1^.min^-1^ to achieve a NOL index of [5-25]. Phenylephrine will be started at 0.2 µg.kg-1.min-1 and adjusted to maintain +/- 20% of the baseline values of the pre-anesthesia mean arterial pressure (MAP). No benzodiazepines or ketamine will be used intraoperatively. An epidural may be placed at the discretion of the anesthesiologist in charge of the patient in the OR. Patients will receive standard prophylaxis against postoperative nausea and vomiting. Hydromorphone PCA (or hydromorphone SC or PO if patient is not a candidate for PCA) for postoperative pain scores < 4/10, or PCEA if an epidural is started in Postoperative anesthesia care unit (PACU). All anesthesia related side effects and quality of analgesia and rehabilitation will be evaluated for 48h.

Intervention

Patients will be randomized (1:1) to receive EEG guided anesthesia versus standard of care. Information provided by the BIS^TM^ (Medtronic, Canada) monitor will guide the volatile anesthetic administration in the EEG-guided group to maintain a BIS value between 40 and 60, a Suppression Ratio (SR; % of time with suppressed brain electrical activity) at 0% or the closest, a direct EEG display without any suppression time and a spectrogram (DSA or density spectral array) with most of the EEG wave frequency within the Alpha (8-12Hz), Theta (4-8Hz) and Delta (0.5-4Hz) frequencies. In the standard of care group, the age-adjusted Minimum Alveolar Concentration (MAC-age) of sevoflurane will be kept at [0.8-1.2] MAC.

Cognitive Assessment

Participants will undergo cognitive assessment preoperatively to establish a baseline and then postoperatively to assess change from that baseline at postoperative days 1, 2, 7, 15, 30 and 90.

- The MoCA or the telephone version of the MoCA (T-MoCA) will be administered at each time point depending on patient location (hospital / home).
- Verbal fluency including phonemic and categorical fluency will be administered at each time point
- The Confusion Assessment Method (CAM) will be used to diagnose postoperative delirium at postoperative days 1 and 2.
- The Quality of Recovery-15 (QoR-15) questionnaire will be used at postoperative day 30 and 90 to explore quality of life after anesthesia and surgery.

Endpoints

An individual reduction of the MoCA score (or T-MoCA depending on testing time point) ≥ 1.96 standard deviation calculated on overall population preoperative mean will define major NCD.

The primary endpoint will be:

- The incidence of major NCD at postoperative day 1.

Secondary endpoints will include:

- the incidence of major NCD at postoperative day 2, 7, 15, 30 and 90
- evolution in cognitive assessment scores over time defining patients’ trajectories,
- the incidence of postoperative delirium during hospital stay,
- the total intraoperative consumption of volatile anesthetics, opioids and vasopressors over surgery time,
- the incidence of intraoperative hypotension
- the incidence of cerebral hypoxemia
- the incidence of awareness,
- cumulative burst suppression duration and low BIS values duration during anesthesia
- the difference between quality of recovery scores between groups.

Tertiary endpoints will include:

• Incidence of major NCD at postoperative day 1 among the following subgroups of patients: different surgical types and duration, patients with preoperative neurocognitive disorder (defined as a preoperative MoCA score < 26), frail patients (defined as a CFS ≥ 5), depressive patients (defined as a PHQ-9 score ≥ 10) and patients above 80 years old.

Significance/Importance

This study was designed to explore the effect of EEG-guided anesthesia on perioperative neurocognitive disorders with tight control of intraoperative blood pressure and nociception level. Understanding EEG patterns of anesthesia to individualize titration of hypnotic drugs may help in reducing the incidence of perioperative neurocognitive disorders, particularly in the elderly.

Study Design

Prospective monocentric randomized controlled trial.

Subject Population

Patients ≥ 70 years old scheduled for major non-cardiac surgery (expected duration ≥1h).

Sample Size

Three hundred and fourteen (314 total; 157 per group, 2 groups) will be included in the present study.

Study Duration

2 years.

Study Center

Single center study, at Maisonneuve-Rosemont Hospital (HMR), CIUSSS de l’Est de l’Ile de Montréal (CEMTL), Montréal, Québec, Canada.

Adverse Events

Non expected.

Funding

Investigator Initiated Trial grant will be submitted to Medtronic Canada in the winter of 2021 (Mars).

Existing research funds of Dr. Philippe Richebé, the PI.

# Introduction

## Summary

With the rapidly expanding aging population, the number of elderly patients undergoing major surgery is increasing. In the province of Quebec, in 2011, the proportion of people above 65 years old was 1 out of 6 (17%) and is expected to be 1 out of 4 (25%) in 2031 (<https://www.inspq.qc.ca/le-vieillissement-au-quebec>) and health related costs are expected to drastically and exponentially increase for patients above 65 years old (ICIS 2011). This presents some major challenges as this cohort has a higher risk of perioperative and postoperative complications. Among these are perioperative neurocognitive disorders, which are linked to extended hospital stays, dependency on social transfer payments, decreased quality of life and increased mortality rates^1^.

Earlier studies reported that electroencephalography (EEG) guided anesthesia can reduce the incidence of both postoperative delirium and postoperative neurocognitive disorders^2-4^. But these studies omitted to control for important factors such as blood pressure, cerebral oximetry level and nociception level (while they may represent confounding factors for the outcome perioperative neurocognitive disorders). More recently, the ENGAGES trial failed to demonstrate any benefit of EEG-guided anesthesia as they avoided intraoperative burst suppression and they only focused on postoperative delirium^5^.

In response to this gap in the literature, we designed a prospective randomized controlled trial (RCT) to explore the impact of EEG-guided anesthesia versus standard of care (SOC – depth of anesthesia based on the age-adjusted MAC of sevoflurane) that included accurately controlled and monitored intraoperative blood pressure and nociception level on NCD evaluated at postoperative day 1 in elderly patients undergoing major non-cardiac surgery.

## Hypothesis

Processed EEG monitors have been used to guide the administration of hypnotic drugs in order to provide optimal depth of anesthesia and avoid overdoses^6^. High doses of anesthetics are directly responsible for neurotoxicity^7^ and increase neuroinflammatory responses to surgical trauma^8^. Excessively deep anesthesia also causes EEG burst suppression that may also alter postoperative neurocognitive functions^9^. Taking into consideration the cardiovascular effects of the anesthetic agents used, EEG-guided anesthesia also may reduce episodes of hypotension or cerebral hypoxemia while preventing intraoperative awareness. We believe that EEG-guided anesthesia may reduce NCD. In order to control for confounding factors our protocol will strictly monitor and manage nociception level, blood pressure and cerebral oximetry with classical algorithms (described in the method section).

# Background

## Prior Literature

### Definition

Perioperative cognitive complications have been reported for more than a century and are common in the elderly^10-12^. These cognitive disorders range from delirium - a fluctuating state of disturbed attention and awareness over a short period of time^13^ - to more subtle and/or longer neurocognitive dysfunctions that may persist for months or years. While these disorders are expected to be transient, some patients will develop longer to permanent cognitive impairments^14^ linked to extended hospital stays, dependency on social transfer payments, decreased quality of life, and increased mortality rates^1^.

It was recently recommended that any cognitive changes observed in the preoperative or postoperative period be categorized under ‘perioperative neurocognitive disorders’^12,15^. Perioperative neurocognitive disorders (NCD) include pre-existing cognitive impairment, postoperative delirium, delayed neurocognitive recovery and postoperative neurocognitive disorders. Delayed neurocognitive recovery refers to neurocognitive disorders from immediate post-operative period until expected recovery from anesthesia and surgery (30 days), while postoperative neurocognitive disorders refer to those from expected recovery time up to 12 months.

### Epidemiology

The first instance of perioperative NCD study was in cardiac surgery^16,17^. The overall incidence of perioperative NCD can reach 70%^18^. Half of patients may develop postoperative delirium after cardiopulmonary bypass^19^. NCD incidence at 1 to 3 months ranges from 10 to 40%^20,21^. Main risk factors and mechanisms were described and classically involve factors such as: age, neuro-inflammation, cardiopulmonary bypass and related micro-embolism^22^.

While the incidence of perioperative NCD in non-cardiac surgery is likely lower, it is still possible. For postoperative delirium, incidence ranges from 5% to 50%^23^. The incidence of NCD is 10% after 3 months, according to the ISPOCD study^24^. Although direct relationship between delirium and NCD remains controversial^25-27^, they certainly share part of their mechanisms, risk factors and long-term importance.

### Risk Factors and Anesthetic Management

Non-modifiable risk factors include age, education level, and pre-existing cognitive impairment^24,26^. As major surgery should only be considered with adequate anesthesia, potential risk factors may arise from the anesthetic management. As such, literature of the last 20 years has focused on exploring different anesthesia-related strategies. For example, regional anesthesia - in comparison with general anesthesia - showed no significant reduction in the incidence of NCD^28^. Several studies have also compared the use of propofol *versus* volatile anesthetic gases for maintenance of general anesthesia. The results are highly controversial and neither agent can be formally implicated in the occurrence of perioperative NCD^29-32^.

Anesthetic adjuvant medications have also been evaluated on perioperative NCD. For example, several studies demonstrated a possible effect of ketamine in reducing NCD after cardiac surgery^33^ while some found no protective effects of ketamine against postoperative delirium^34^. Dexamethasone was found to decrease the rate of NCD in elderly patients when administered in single dose at induction of general anesthesia. But this result was only observed in a subgroup of patients with a relatively higher Bispectral index (BIS)^35^.

### Anesthesia Depth

The Bispectral index (BIS) measures anesthesia depth. BIS values can range from 0 to 100 where lower values correspond to deeper anesthesia. Due to the lack of explicit monitoring results, three trials investigated the effect of BIS monitoring on postoperative NCD incidence^2-4^. Ballard et al. showed that EEG and cerebral oximetry monitoring reduced the rate of mild neurocognitive disorders at 1 week and 3 months after surgery compared with routine care^2^. There was no effect on severe NCD but the study sample was low (72 patients total). The CODA trial compared 921 patients randomly assigned to receive BIS-guided anesthesia or routine care^3^. Patients in the routine care group had a higher rate of NCD at 3 months than BIS-guided patients. This higher rate of NCD was associated with lower BIS, longer duration of BIS < 40 and higher anesthetic dose administered in the routine care group. No difference in the rate of NCD at 1 week was observed among groups. Radtke et al. studied more than 1100 patients randomly allocated to have BIS monitoring versus standard of care^4^. The rate of NCD at 1 week and 3 months (explored as secondary endpoints) were not different between groups. But, in contrast to CODA, mean BIS values were similar in the BIS and control groups.

Nevertheless, these three previous studies had strong limitations: blood pressure management data were unavailable (low blood pressure during surgery may clearly be a cofounding factor for NCD^36,37^) and nociception levels were never monitored, ignoring the effect intraoperative large doses of opioids or intraoperative excess of nociception might also have on postoperative NCD. Finally, results were unclear if reduction in perioperative NCD incidence was related to avoidance of burst suppression – an electroencephalographic pattern suggesting excessively deep anesthesia^38^.

In 2020, a multidisciplinary organization – the Perioperative quality initiative (POQI) - developed consensus-based recommendations on the association between intraoperative processed EEG monitoring and postoperative NCD^39^. They recommended clinicians be knowledgeable in EEG interpretation (raw waveform, spectrogram, and processed indices) when using these technologies in anesthetic management and using EEG monitoring to detect unintended burst suppression during general anesthesia. They also carried out a meta-analysis of the 3 above-mentioned studies. The pooled analysis showed a significant decrease in the risk of developing postoperative NCD with BIS monitoring. Nevertheless, until further data are available, they concluded that there remains insufficient evidence to recommend the routine use of EEG monitoring for the prevention of postoperative NCD.

## Study Rationale

Since 2013 and the last study by Radtke et al.^4^, the literature on EEG-guided anesthesia has mostly focused on postoperative delirium^5^. To our knowledge, no major study has been published since the new nomenclature on neurocognitive disorders appeared^15^. In addition, processed EEG monitors have become more sophisticated, allowing, among other new parameters, to display the spectral density array (DSA) extracted from the raw EEG, the burst suppression ratio and the total burst suppression time. The clinician can now rely on multiple pieces of information to conduct the patient’s hypnosis. These are crucial since EEG under general anesthesia with propofol and volatile anesthetic gases varies with age^40^ and burst suppression may be associated with NCD.

Our objective here is to explore the effect of EEG-guided anesthesia on neurocognitive disorders compared to standard of care (SOC).

In line with recent recommendations^39^, we propose to use all the information provided by the BIS^TM^ (Medtronic, Canada) processed EEG monitor including the BIS value, the raw waveform, the spectrogram and the suppression ratio to guide anesthesia in the EEG-guided group. The use of this type of information is common practice in our center.

Since opioid use may introduce a bias when observing for perioperative NCD, we will use a strict protocol **for the control of nociception level and intraoperative opioid infusion**. The PMD-200^TM^ monitor (Medasense Biometrics, Ramat Gan, Israel) is a novel monitor that measures nociception level via a combination of multiple physiological parameters (heart rate, heart rate variability, plethysmograph wave amplitude, skin conductance, and skin conductance variation)^41-43^. The Nociception level (NOL) index is the value calculated by the PMD-200^TM^ monitor and ranges from 0 to 100 where optimal analgesia is between 5 and 25. The NOL index is a tested and validated measure that showed a high-correlation between pain measurements and doses of intraoperative opioids^44,45^ and it is commonly used in our center at Maisonneuve-Rosemont hospital / CEMTL.

We will also strictly **control arterial blood pressure and cerebral oximetry** to avoid the risk of low blood pressure and cerebral hypoxemia during surgery and their possible effect on our postoperative outcomes. We will calculate targets for mean arterial pressure (MAP) during surgery from three pre-anesthesia blood pressure measurements and monitor bilateral cerebral oximetry.

In a previous study, we evaluated as an exploratory outcome NCD in a population undergoing major colorectal surgery under general anesthesia. We established the incidence of major NCD at postoperative day 1 was 3.8% in the monitored group (EEG-guided anesthesia with BIS index) versus 15.4% in the control group (standard of care group, BIS placed but not used to guide the intraoperative administration of the halogenous gas). We therefore chose to use the incidence of NCD at postoperative day 1 as a primary objective in the proposed study.

# Study Objectives

## Primary Objective

The main objective of the study is to investigate whether EEG-guided anesthesia – intended to reduce anesthetic administration and minimize burst suppression during general anesthesia – **decreases the incidence of NCD at postoperative day 1 in elderly patients (> 70 years old)** undergoing major (expected duration > 1h) non-cardiac surgery compared to standard of care (SOC, BIS installed but not used to guide anesthesia).

## Secondary Objectives

Secondary objectives are to investigate the effect of EEG-guided anesthesia on:

- neurocognitive disorders (NCDs) at postoperative day 2, 7, 15, 30 and 90,
- patients’ perioperative cognitive trajectories,
- postoperative delirium,
- intraoperative consumption of volatile anesthetics, opioids and vasopressors,
- intraoperative hypotension (number of interventions to treat hypotensive events),
- cerebral hypoxemia (assessed by cerebral oximetry),
- cumulative burst suppression duration and cumulative low processed EEG (BIS) values duration during anesthesia,
- risk of awareness,
- postoperative quality of life / recovery outcomes (including length of hospital stay and surgical complications).

## Tertiary objectives

Tertiary objectives are to:

- explore the effect of EEG-guided anesthesia compared to standard of care across and within subgroups including different categories of surgical, duration of surgery, preoperative frailty, preoperative presence of depressive symptoms, age and patients with preoperative neurocognitive disorder.

# Methods

## Study design

This study will be a prospective monocentric randomized controlled trial. Patients will be randomly assigned to the one of the two groups (EEG-guided with BIS *versus* standard of care – SOC). Processed EEG (BIS) will be monitored in both groups but information from those monitors will only be available for the EEG-guided group to guide hypnotic drugs’ administration. All other aspects of anesthesia will be the same between the groups, especially concerning the management of nociception and blood pressure.

## Subject population

All consecutive patients ≥ 70 years old who are scheduled for major gynecologic, abdominal, urologic, thoracic or orthopedic surgery and seen for assessment by internal medicine and/or anesthesiology at the preoperative clinic (CIEPC).

### Inclusion criteria

- Patients 70 years of age or older,
- Major gynecologic, abdominal, urologic, thoracic or orthopedic surgery via laparoscopy or laparotomy under general anesthesia - with or without concomitant use of regional or neuraxial anesthesia -,
- Expected anesthesia time of more than 60 minutes,
- Seen for assessment by internal medicine and/or anesthesiology at the preoperative clinic (CIEPC)

### Exclusion criteria

- Known diagnosis of dementia or other neurological, psychiatric, developmental or medical condition that resulted in documented severe cognitive impairment,
- Emergency surgery,
- Significant auditory or visual impairment that precludes participation in cognitive testing,
- Known allergy or intolerance or other medical condition that precludes the use of prescribed general anesthesia protocol for this study,
- Inability to communicate in French or English.

### Recruitment

All patients will be assessed and recruited by internal medicine and/or anesthesiology from the preoperative clinic (CIEPC) in collaboration with the research team for this study, informed of the study parameters, and provided a consent form to be signed. A total of 314 patients (157 per group) will be included in the study (see below). We estimate an inclusion rate of 3 to 5 patients per week (study duration for inclusions 2 years). In order to avoid missing data, we will conduct telephone interviews for cognitive assessment of patients once they return home.

## Research plan and timeline

Patients will be screened at the preoperative clinic. The day of the surgery (D0), after signing the consent form, patients will undergo the inclusion visit. Cognitive assessment will be performed using the MoCA and verbal fluency tests by a trained research team. Patients will also be screened for depression and frailty. The patient will then be randomized in the EEG-guided or SOC group.

Postoperative visit will be performed at postoperative days 1 and 2 (D1 and D2). Cognitive assessments will be performed using MoCA and verbal fluency tests. Patients will also be screened for postoperative delirium.

Once patients return home, phone interviews will be done at D7, D15, D30 and D90. Cognitive assessments will be performed using the telephone version of the MoCA (T-MoCA) and verbal fluency tests. Quality of recovery will be evaluated at D30 and D90 using the QoR-15 questionnaire.

T-MoCA and T-MoCA part of the MoCA as well as verbal fluency will be used to establish cognitive trajectories of the two groups.

The 3 versions of the MoCA will be alternately and randomly used to limit learning effect.

Estimated time for each visit is approximatively 45 minutes. The study timeline used tests and expected cognitive trajectories are presented in figure 1.

All the tests are provided in the references and detailed below. These tests are also attached in the annexes in French and in English.


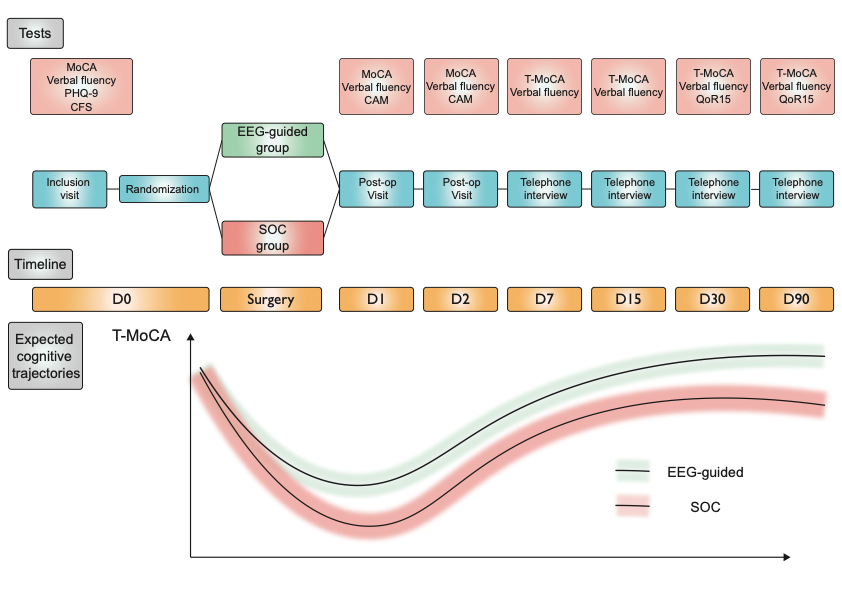


**Figure 1. Study timeline, tests and expected cognitive trajectories**. *MoCA: Montréal Cognitive Assessment; PHQ-9: Patient Health Questionnaire 9; CFS: Clinical Frailty Scale; EEG: electroencephalograph; SOC: Standard of Care; CAM: Confusion Assessment Method; QoR: Quality of Recovery; T-MoCA: Telephone MoCA.*

## Anesthesia Protocol

### General

All patients will undergo general anesthesia with the following: induction with IV slow boluses of lidocaine to numb the vein, propofol 1.5mg.kg^-1^, remifentanil 1µg.kg^-1^ over 30 seconds, rocuronium 0.8 mg.kg^-1^. We will proceed to orotracheal intubation when response to neuromuscular stimulation of the adductor pollicis has reached 5% of the original T4 amplitude. The maintenance of anesthesia will depend on sevoflurane to reach [0.8–1.2] minimal alveolar concentration (MAC; MAC adjusted to the patient’s age) in the control group, and to achieve a BIS of [40-60] in the EEG-guided group (see below). The remifentanil infusion will start at 0.02 µg.kg^-1^.min^-1^ and increase by 0.02 µg.kg^-1^.min^-1^. If the NOL index is > 25 for more than 1 min, we will administer one bolus of 0.3 µg.kg^-1^. The maximum remifentanil rate allowed intraoperatively will be 0.3 µg.kg^-1^.min^-1^. This rate will be decreased by step of 0.02 µg.kg^-1^.min^-1^ if the NOL index is < 5 for more than 3 minutes (for a minimum rate of 0.02 µg.kg^-1^.min^-1^). We will administer intravenous boluses of rocuronium as needed for the response to TOF stimulation to stay below 2/4.

Baseline mean arterial blood pressure (MAP) will be defined as the average of 3 consecutive values taken 1 min apart and determined before the induction of general anesthesia. Intravenous (IV) infusion of phenylephrine will start at 0.2 µg.kg^-1^.min^-1^ and adjusted to maintain +/- 20% of the baseline values of the pre-anesthesia MAP. Benzodiazepines or ketamine will not be used, and patients will receive standard prophylaxis against postoperative nausea and vomiting (avoiding haloperidol).

Remifentanil and phenylephrine infusions will be discontinued when the skin is closed. At the same time, muscle relaxation will be antagonized using neostigmine 0.04 mg.kg^-1^ and glycopyrrolate 0.01 mg.kg^-1^, and bolus of ondansetron 4mg and hydromorphone 0.006 µg.kg^-1^ administered. Sevoflurane will be discontinued when the wound dressing is completed, and patients will be extubated in the operating room, then transferred to the post-anesthesia care unit (PACU).

Hydromorphone PCA (or SC hydromorphone if patient is not a candidate for PCA) for postoperative pain scores < 4/10, or PCEA (if an epidural was placed) is started in PACU. All patients will be managed according to ERAS (Enhanced recovery after surgery) principles. All anesthesia related side effects and quality of analgesia and, rehabilitation will be evaluated for 48h (see below).

### Monitoring

Routine monitoring of patients will be the same for both groups and will include 5 leads ECG, non-invasive blood pressure, pulse-oximetry and a temperature probe. We will record all data using the Dräger Perseus A500 (Dräger Medical, Lübeck, Germany) monitor and send all anesthesia related data electronically to a research computer for anonymized storage and further analysis.

We will place the NOL index finger probe (PMD-200™ device, Medasense Biometrics Ltd, Ramat Gan, Israel) and the bilateral cerebral regional oximetry rSO_2_ (Invos™, Medtronic, Canada) (both are available to the anesthesiologist for both groups throughout the entire anesthesia).

Bilateral BIS^TM^ EEG sensor (Medtronic, Canada) will be placed on the forehead for all patients. For the SOC (standard of care) group, only the electrode status display will be available; the rest of the data will be hidden by a dark screen – and unavailable to the anesthesiologist during the anesthesia.

### Intervention

In the EEG-guided group, the information provided by the BIS^TM^ monitor will guide the volatile anesthetic administration in the EEG-guided group. Sevoflurane end-tidal anesthetic concentration (ETAC) will be adjusted to maintain a BIS value between 40 and 60, a Suppression Ratio (SR; % of time with suppressed brain electrical activity) at 0%, a direct EEG display without any suppression time and a spectrogram (DSA or density spectral array) with most of the EEG wave frequency within the Alpha (8-12Hz), Theta (4-8Hz) and Delta (0.5-4Hz) frequencies.

In the non-EEG-guided group, the MAC of sevoflurane (adjusted to age) will be used to guide the intraoperative anesthesia delivery and MAC will be kept between 0.8 and 1.2.

## Randomization and blinding

A randomization list will be electronically made and followed for the 314 subjects. An envelope containing the study group will be given to the anesthesiologist in charge once the patient will be in the operating room and be opened after the inclusion visit to limit contamination bias. All patients and researchers assessing outcomes and adverse events will be blinded to randomization assignments.

## Measurements

### Cognitive Assessments

#### The Montreal Cognitive Assessment tool

Participants will undergo preoperative cognitive assessment to establish a baseline, then postoperatively to assess changes from that baseline. We will perform preoperative assessment the morning of the scheduled surgery and postoperative assessments on days 1, 2, 7, 15, 30 and 90.

All cognitive assessments will use the Montreal Cognitive Assessment (MoCA) tool. The MoCA takes roughly 10 min to administer and covers eight cognitive domains. It was originally designed as a screening tool to detect mild cognitive impairment (MCI)^46^ but it is also used in clinical and research assessment of a variety of neurological and cognitive pathologies. A score below 26 is considered positive for cognitive impairment. It has three versions, allowing for retesting with low risk of learning between assessments. The telephone version of the MoCA (T-MoCA) is scored on a scale of 0 to 22 and corresponds to the MoCA and excludes the visuospatial/executive and naming component. The T-MoCA has proved to be a reliable method to assess MCI after stroke^47^ and has been used to explore cognitive functions after major non-emergent surgery^27^. As mentioned in the exclusion criteria, patients with preoperative documented diagnosis of dementia or other neurological, psychiatric, developmental or medical condition that resulted in documented severe cognitive impairment will not be included in the proposed study. Nevertheless, in the event that we diagnose a preoperative neurocognitive disorder (i.e., preoperative MoCA <26), patients will still be included in the study. At the end of the follow-up (at 3 months postoperatively), patients will be informed of the possibility of an incipient neurocognitive disorder and referred to a neuropsychologist for a more thorough neuropsychological test battery. Subgroup analyses are planned for patients with preoperative neurocognitive impairment

In our previous feasibility study, we encountered difficulties administering MoCA to patients who had left the hospital. So, we chose to use MoCA for in-hospital cognitive assessments and the T-MoCA once patients returned home. The MoCA scores will be used for the primary endpoint assessment – pre-anesthesia versus at H24 after surgery- and T-MoCA scores will be used to establish the cognitive trajectories up to 3 months after general anesthesia for major surgery in this elderly population.

#### Verbal Fluency

Testing verbal fluency tests requires a participant to generate as many words as possible within set times and parameters - phonemic verbal fluency: *e.g.,* words that start with the letter f, categorical verbal fluency: *e.g.,* names of animals. These verbal tasks evaluate lexical access but also assess sustained attention and memory. These tests can be completed in person or over the phone – both have proven reliable means of administering the test in both French and English^48-51^ – and are excellent tools to assess a variety of cognitive impairments across many disorders (MCI, Alzheimer Dementia, traumatic brain injury, Parkinson Disease).

#### Z Scores and Definition of Neurocognitive Disorders (NCD)

We will calculate Z scores for individual tests - (Verbal fluency, MoCA, T-MoCA) at each testing time point with the mean and standard deviation (SD) of baseline tests of all patients - in the following way:

$$Z_{test}=\frac{x_{test}-\mu_{test baseline}}{\sigma_{test baseline}}$$

where $x$ corresponds to individual test value, $\mu$ and $\sigma$ correspond to test mean and SD of all patients at baseline respectively.

We will use MoCA Z scores (or T-MoCA depending on the testing time point) to define NCD. Neurocognitive disorders will be classified as mild or major^12,15^:

- **No NCD**: a decrease in Z score < 1 SD,
- **Mild:** a decrease in Z score ≥ 1 SD,
- **Major:** a decrease in Z score ≥ 1.96 SD.

| Z score decrease | Postoperative days 1,2,7,15, 30 and 90 |
| --- | --- |
| < 1 SD | No NCD |
| ≥ 1 SD and < 1.96 SD | Mild NCD |
| ≥ 1.96 SD | Major NCD |

### Other Assessments

#### Preoperative Assessment of Depression and Frailty

We will pre-screen for symptoms of severe depression and extreme fragility at preoperative enrollment, to ensure cognitive impairment is not related these two conditions. We will use the Patient Health Questionnaire 9 (PHQ-9) to detect severity of depression^52^ and the Clinical Frailty Scale 2.0^53^ (CFS) to assess frailty. Both screening tools are easy to use for clinicians, are validated, and available in French^54,55^.

#### Delirium Assessment

We will administer the Confusion Assessment Method (CAM) on postoperative days 1 and 2. The CAM is a validated assessment tool for the diagnosis of delirium^56^ and is based on the diagnostic criteria for delirium from the Diagnostic and Statistical Manual of Mental Disorders (DSM). The cognitive testing on postoperative days 1 and 2 will consist of basic orientation questions and a sustained attention task (stating the months of the year backwards). If the CAM yields a positive diagnosis, we will administer a subsequent test, the CAM-S which assesses the severity of delirium. In the case of persistent mechanical ventilation after surgery in the ICU, we will administer the Intensive Care Unit version of the CAM (CAM-ICU)^57^.

#### Postoperative Assessment of Quality of Life

We will perform the Quality of Recovery-15 (QoR-15) interview will be performed at postoperative day 30 and 90 to explore quality of life after anesthesia and surgery, and to assess differences between groups. This is a validated shorter version of the QoR-40 with only 15 items^58^.

### Endpoints

#### Primary Endpoint

- Incidence of major NCD at postoperative day 1.

#### Secondary Endpoints

- The incidence of major NCD at postoperative day 2, 7, 15, 30 and 90
- The evolution over time in cognitive assessment scores defining cognitive trajectories,
- The incidence of postoperative delirium during hospital stay,
- The total intraoperative consumption of volatile anesthetics, opioids and vasopressors over surgery time
- The incidence of intraoperative hypotension,
- The incidence of cerebral hypoxemia,
- The cumulative burst suppression duration and low BIS values duration during anesthesia,
- The incidence of awareness,
- The difference between quality of recovery scores between groups.

#### Tertiary endpoints

- Incidence of major NCD at postoperative day among the following subgroups of patients: different surgical types and duration, patients with preoperative neurocognitive disorder (defined as a preoperative MoCA score < 26 ^46^), frail patients (defined as a CFS ≥ 5 ^59^), depressive patients (defined as a PHQ-9 score ≥ 10 ^60^) and patients above 80 years old.

# Subject Visits

## Data Collection Procedures

We will collect all electronic data from the medical monitoring including BIS^TM^ and PMD-200^TM^. Monitor times will be synchronized before any data is collected. We will note on a separate Case Report Form (CRF) and in the integrated system of PMD-200^TM^, all anesthesia and surgery related events. At the end of anesthesia, and once the patient is extubated, we will export all electronic data. Data will be anonymized and stored in a dedicated research computer at the Department of Anesthesiology and Pain Medicine of HMR-CEMTL.

## Safety and Adverse Events

Research personnel and hospital staff will make all efforts to ensure the safety of the participants. We expect no adverse events resulting from this research.

### Anesthesia Management Protocol

The anesthetic management in the proposed study represents a standard and conventional approach to general anesthesia (see “Risks” section below). There is no additional anesthesia-related patient risk arising from participation in this study.

### Cognitive Assessment

During recruitment, we will explain to all patients that participation in the study is voluntary, non-binding, and that cognitive assessment performance has no bearing on medical treatment. The cognitive tests used in the proposed study will require the participant to verbally interact with the examiner and, for a few tasks, write words or draw images – the risk of physical harm from testing is minimal or not existing. We will schedule (to the best of our ability) postoperative testing when patients are awake, comfortable, and not slated for any treatments (dressing changes, physiotherapy, etc.).

### Medical Monitoring

The PI will review all data, including completeness of study data, enrolment, protocol deviations, dropouts, adverse events on a regular basis (weekly or bi-weekly) and an annual report of all adverse events. A summary of the investigation will be submitted to the approving Internal Review Board (IRB).

### Definition of Adverse Events

There are no anticipated adverse events for this research. If adverse events occur, the PI research personnel will report it to the approving IRB. Adverse events will be graded as: Mild, Moderate, or Serious; and Related, Possibly Related, or Not Related to study procedures.

Adverse Event: Any unfavorable and unintended sign, symptom or disease temporarily associated with the use of a medical treatment or procedure.

Serious Adverse Event: Any adverse event that results in any of the following outcomes:

- Death
- Outpatient hospitalization or prolongation of existing hospitalization
- Persistent or significant disability/incapacity.

# Proper Handling of Subjects

## Ethical Considerations

Cognitive testing may reveal undiagnosed cognitive impairments.

## Subject Withdrawal

### Early Withdrawal of Subjects

We will track the number of withdrawn subjects. Possible reasons a subject may withdraw are a desire to discontinue their participation, or a deterioration in clinical condition or level of consciousness to the point where the patient can no longer participate.

### When and How to Withdraw Subjects

A withdrawal occurs when a subject voluntarily chooses to leave or is no longer able to participate in the research study. We will remove a subject if they mention to the research personnel that they no longer wish to continue with the study or when the research personnel note that, for medical reasons, the subject is no longer able to participate. The research personnel will keep all personal health information secured in the database.

### Data Collection and Follow-up for Withdrawn Subjects

We will not store data or follow-up after subject withdrawal.

## Risks

There is very little added risk involved with participation in this study and the protocol is not expected to increase duration of anesthesia. The anesthetic care provided will be routine, we will use standard intraoperative monitoring, and only use common analgesics, such as intravenous remifentanil.

# Statistical Plan

## Sample Size

In a previous study at HMR/CEMTL, we evaluated as an exploratory outcome NCD in a population (mean age of 65) undergoing major colorectal surgery under general anesthesia. We established the incidence of major NCD (using pre- *versus* 24h after surgery MMSE testing; Z score ≥ 1.96 SD in MMSE evaluations pre and postoperatively) at postoperative day 1 was 3.8% in the monitored group (EEG-guided anesthesia with BIS index) versus 15.4% in the control group (standard of care group, BIS placed but not used to guide intraoperative administration of the halogenous gas). Similar results had also been observed by Hou R. et al. in 2018^61^. Based on a 2-sided α <0.05 and 80% power, we calculated that the enrolment of 282 patients was required to detect (as the primary endpoint of the proposed study) a clinically significant and relevant reduction of at least reduction of 10% of NCD incidence in the EEG-guided group compared to the control group (expected decrease from 15% to 5% or less). The sample size will be inflated to 314 (157 in each group) to account for 10% withdrawals and loss of follow-up.

## Statistical Methods

### General Considerations

Descriptive statistics will be presented by groups using mean (SD) or median (Q1, Q3) for continuous parameters (according to the skewness or not of the distribution of each parameter), and frequency (%) for categorical parameters. According to the nature of the analyzed endpoints, confidence intervals (CIs) between proportions or between means/medians differences will be presented. The alpha value will be set a 0.05. All data will be analyzed by the intention to treat principle. All statistical analysis will be performed using SAS, SPSS or R software.

### Primary Analysis

Incidence of NCD will be presented using frequency and proportion (%), broken down by groups (EEG use or SOC). The distribution of presence or absence of NCD will be compared between the two groups using a chi-square test. A risk difference will be reported with a score (Wilson) 95% confidence interval.

In a case we observe important quantitative imbalance between prognostic factors between the treatment groups, we will conduct an adjusted analysis of the primary outcome. We will estimate a standardized marginal average treatment effect (risk difference) model adjusted from 10 strata based on an estimated propensity score from NCD risk factors (age, sex, preoperative MoCA score, CFS, PHQ-9 and education level). The marginal risk difference will be reported with 95% confidence intervals, estimated by non-parametric bootstrap (10 000 replications).

### Secondary Analysis

Presentation of results and group comparisons (EEG use or SOC) for the secondary dichotomous endpoints will be done using the same approach described in the primary analysis. For the continuous endpoints, the group comparisons will be done using a t-test or a Mann-Whitney-Wilcoxon test according to the distribution of each parameters and a chi-square test will be used for proportion. Risk differences or mean differences with 95% confidence intervals will be reported for all outcomes.

### Trajectories Analysis

Z scores of T-MoCA and T-MoCA part of the MoCA test (baseline, D1, D2, D7, D15, D30 and D90) will be entered in a group-based trajectory model (GBTM) to derive trajectories of cognitive function. GBTM allows for the identification of patient subgroups sharing similar cognitive patterns over time^62,63^. Different models will be tested that vary based on the number of trajectories (models that contain between 1 and 8 trajectories will be tested) and inclusion of linear and quadratic terms. Selection of the final model is based on Bayesian Information Criteria (BIC), parsimony, and ≥ 5% of patients in each class. Once the final model is selected, outcome variables represent probabilities of belonging to each of the trajectories. For each of the relevant baseline variables, the GBTM obtained will be rerun with the inclusion of age, sex, randomization group and education as covariates. For each model, an odds ratio will allow for the examination of the contribution of the variable of interest to the classification of patients in each trajectory.

### Subgroups and sensitivity analyses

Subgroups analyses will be conducted in the following subgroups: different surgical types and duration, patients with preoperative neurocognitive disorder (defined as a preoperative MoCA score < 26), frail patients (defined as a CFS ≥ 5), depressive patients (defined as a PHQ-9 score ≥ 10) and patients above 80 years old. Stratified treatment effects (risk difference of mean difference) will be reported for our primary outcome with 95% confidence intervals within each subgroup as well as the result of an interaction test between subgroup characteristic and allocated treatment.

# Data Handling and Record Keeping

## Confidentiality and Security

Protected health information will not be re-used by our institution or disclosed to a third party - except as required by law, for authorized oversight of the research, or as permitted by written authorization signed by the research subject.

## Electronic Data

Data will be stored on a password-protected laptop which has been assigned to Dr. Philippe Richebé, who will maintain primary responsibility for this computer and the data.

## Hard Data

Paper copies (consent forms) of patient information will be stored in a locked file cabinet and office in the Department of Anesthesiology and Pain Medicine.

## Coding Data

Data are confidential. We will assign data unique study codes (P1, P2, and so on up until PX) that link to the subject’s identity. This link will be kept separately to avoid patient identification in the event of theft or loss of data.

## Accessibility

Only the PI will have access to all the data during the study.

## Training

Every person involved in this study will receive appropriate training, abide by confidentiality guidelines to protect the subject’s privacy, strictly follow rules and guidelines, as outlined in the Health Information Protection Act (Canada).

## Linked Data

There will be no linked data.

# Study Monitoring, Auditing and Inspecting

## Study Monitoring Plan

Every month the PI will review the recruitment and data collection.

## Auditing and Inspecting

There will be no formal plan for audit or inspection.

# Study administration

## Organization and Recruiting Site, IRB

**PI:** Philippe Richebé, MD, PhD, Full Professor,

**Research coordinator:** Nadia Godin, RN, Nurse and Research Coordinator.

**Research assistant:** Moulay Idrissi, BEng, MSc.

**Recruiting Site:** Maisonneuve-Rosemont Hospital, CIUSSS de l’Est de l’Ile de Montreal, University of Montréal.

## Study Timetable

IRB submission: March 11^th^, 2021

Beginning of inclusion: April-May 2021

Estimated ending of inclusion: Spring 2023 (3-5 inclusions per week)

Data analysis: Summer 2023

Publication: Summer 2023.

# Publication Plan

Publication of the study protocol will be submitted once approved by the Ethical committee to the journal PLoS ONE.

Publication of the results of the present study will be proposed to JAMA or Lancet, and if not accepted to ANESTHESIOLOGY or British Journal of Anaesthesia.

# Budget

This study will be supported by funds from Dr Richebé Philippe at the CR-HMR and the Department of Anesthesiology and Pain Medicine. An application as an Independent Investigator Initiated Trial grant will be submitted to Medtronic for about 318 000 CA$ including all overhead costs for the CR-HMR (Research center of HMR/CEMTL, University of Montreal) in the first trimester of 2021.

# References

1. Steinmetz J, Christensen KB, Lund T, Lohse N, Rasmussen LS, Group I. Long-term consequences of postoperative cognitive dysfunction. *Anesthesiology.* 2009;110(3):548-555.

2. Ballard C, Jones E, Gauge N, et al. Optimised anaesthesia to reduce post operative cognitive decline (POCD) in older patients undergoing elective surgery, a randomised controlled trial. *PLoS One.* 2012;7(6):e37410.

3. Chan MT, Cheng BC, Lee TM, Gin T, Group CT. BIS-guided anesthesia decreases postoperative delirium and cognitive decline. *J Neurosurg Anesthesiol.* 2013;25(1):33-42.

4. Radtke FM, Franck M, Lendner J, Kruger S, Wernecke KD, Spies CD. Monitoring depth of anaesthesia in a randomized trial decreases the rate of postoperative delirium but not postoperative cognitive dysfunction. *Br J Anaesth.* 2013;110 Suppl 1:i98-105.

5. Wildes TS, Mickle AM, Ben Abdallah A, et al. Effect of Electroencephalography-Guided Anesthetic Administration on Postoperative Delirium Among Older Adults Undergoing Major Surgery: The ENGAGES Randomized Clinical Trial. *JAMA.* 2019;321(5):473-483.

6. Punjasawadwong Y, Phongchiewboon A, Bunchungmongkol N. Bispectral index for improving anaesthetic delivery and postoperative recovery. *Cochrane Database Syst Rev.* 2014(6):CD003843.

7. Wan Y, Xu J, Meng F, et al. Cognitive decline following major surgery is associated with gliosis, beta-amyloid accumulation, and tau phosphorylation in old mice. *Crit Care Med.* 2010;38(11):2190-2198.

8. Vacas S, Degos V, Feng X, Maze M. The neuroinflammatory response of postoperative cognitive decline. *Br Med Bull.* 2013;106:161-178.

9. Fritz BA, Kalarickal PL, Maybrier HR, et al. Intraoperative Electroencephalogram Suppression Predicts Postoperative Delirium. *Anesth Analg.* 2016;122(1):234-242.

10. Savage GH. Insanity following the Use of Anæsthetics in Operations. *Br Med J.* 1887;2(1405):1199-1200.

11. Bedford PD, Leeds MD. Adverse cerebral effects of anaesthesia on old people. *The Lancet.* 1955;266(6884):259-264.

12. Evered L, Silbert B, Knopman DS, et al. Recommendations for the Nomenclature of Cognitive Change Associated with Anaesthesia and Surgery-2018. *Anesthesiology.* 2018;129(5):872-879.

13. American Psychiatric Association, DSM-5, Diagnostic and Statistical Manual of Mental Disorders, fifth edition. 2013.

14. Inouye SK, Marcantonio ER, Kosar CM, et al. The short-term and long-term relationship between delirium and cognitive trajectory in older surgical patients. *Alzheimers Dement.* 2016;12(7):766-775.

15. Evered L, Silbert B, Knopman DS, et al. Recommendations for the nomenclature of cognitive change associated with anaesthesia and surgery-2018. *Br J Anaesth.* 2018;121(5):1005-1012.

16. Shaw PJ, Bates D, Cartlidge NE, et al. Early intellectual dysfunction following coronary bypass surgery. *Q J Med.* 1986;58(225):59-68.

17. Shaw PJ, Bates D, Cartlidge NE, et al. Long-term intellectual dysfunction following coronary artery bypass graft surgery: a six month follow-up study. *Q J Med.* 1987;62(239):259-268.

18. Newman MF, Mathew JP, Grocott HP, et al. Central nervous system injury associated with cardiac surgery. *Lancet.* 2006;368(9536):694-703.

19. Brown CHt, Probert J, Healy R, et al. Cognitive Decline after Delirium in Patients Undergoing Cardiac Surgery. *Anesthesiology.* 2018;129(3):406-416.

20. Ottens TH, Dieleman JM, Sauer AM, et al. Effects of dexamethasone on cognitive decline after cardiac surgery: a randomized clinical trial. *Anesthesiology.* 2014;121(3):492-500.

21. Mathew JP, White WD, Schinderle DB, et al. Intraoperative magnesium administration does not improve neurocognitive function after cardiac surgery. *Stroke.* 2013;44(12):3407-3413.

22. Berger M, Terrando N, Smith SK, Browndyke JN, Newman MF, Mathew JP. Neurocognitive Function after Cardiac Surgery: From Phenotypes to Mechanisms. *Anesthesiology.* 2018;129(4):829-851.

23. Rudolph JL, Marcantonio ER. Review articles: postoperative delirium: acute change with long-term implications. *Anesth Analg.* 2011;112(5):1202-1211.

24. Moller JT, Cluitmans P, Rasmussen LS, et al. Long-term postoperative cognitive dysfunction in the elderly ISPOCD1 study. ISPOCD investigators. International Study of Post-Operative Cognitive Dysfunction. *Lancet.* 1998;351(9106):857-861.

25. Franck M, Nerlich K, Neuner B, et al. No convincing association between post-operative delirium and post-operative cognitive dysfunction: a secondary analysis. *Acta Anaesthesiol Scand.* 2016;60(10):1404-1414.

26. Saczynski JS, Marcantonio ER, Quach L, et al. Cognitive trajectories after postoperative delirium. *N Engl J Med.* 2012;367(1):30-39.

27. Austin CA, O'Gorman T, Stern E, et al. Association Between Postoperative Delirium and Long-term Cognitive Function After Major Nonemergent Surgery. *JAMA Surg.* 2019;154(4):328-334.

28. Silbert BS, Evered LA, Scott DA. Incidence of postoperative cognitive dysfunction after general or spinal anaesthesia for extracorporeal shock wave lithotripsy. *Br J Anaesth.* 2014;113(5):784-791.

29. Cai Y, Hu H, Liu P, et al. Association between the apolipoprotein E4 and postoperative cognitive dysfunction in elderly patients undergoing intravenous anesthesia and inhalation anesthesia. *Anesthesiology.* 2012;116(1):84-93.

30. Enlund M, Mentell O, Flenninger A, Horneman G, Ronquist G. Evidence of cerebral dysfunction associated with isoflurane- or propofol based anaesthesia for orthognathic surgery, as assessed by biochemical and neuropsychological methods. *Ups J Med Sci.* 1998;103(1):43-59.

31. Royse CF, Andrews DT, Newman SN, et al. The influence of propofol or desflurane on postoperative cognitive dysfunction in patients undergoing coronary artery bypass surgery. *Anaesthesia.* 2011;66(6):455-464.

32. Schoen J, Husemann L, Tiemeyer C, et al. Cognitive function after sevoflurane- vs propofol-based anaesthesia for on-pump cardiac surgery: a randomized controlled trial. *Br J Anaesth.* 2011;106(6):840-850.

33. Hudetz JA, Pagel PS. Neuroprotection by ketamine: a review of the experimental and clinical evidence. *J Cardiothorac Vasc Anesth.* 2010;24(1):131-142.

34. Avidan MS, Maybrier HR, Abdallah AB, et al. Intraoperative ketamine for prevention of postoperative delirium or pain after major surgery in older adults: an international, multicentre, double-blind, randomised clinical trial. *Lancet.* 2017;390(10091):267-275.

35. Valentin LS, Pereira VF, Pietrobon RS, et al. Effects of Single Low Dose of Dexamethasone before Noncardiac and Nonneurologic Surgery and General Anesthesia on Postoperative Cognitive Dysfunction-A Phase III Double Blind, Randomized Clinical Trial. *PLoS One.* 2016;11(5):e0152308.

36. Sessler DI, Sigl JC, Kelley SD, et al. Hospital stay and mortality are increased in patients having a "triple low" of low blood pressure, low bispectral index, and low minimum alveolar concentration of volatile anesthesia. *Anesthesiology.* 2012;116(6):1195-1203.

37. Willingham M, Ben Abdallah A, Gradwohl S, et al. Association between intraoperative electroencephalographic suppression and postoperative mortality. *Br J Anaesth.* 2014;113(6):1001-1008.

38. Amzica F. Basic physiology of burst-suppression. *Epilepsia.* 2009;50 Suppl 12:38-39.

39. Chan MTV, Hedrick TL, Egan TD, et al. American Society for Enhanced Recovery and Perioperative Quality Initiative Joint Consensus Statement on the Role of Neuromonitoring in Perioperative Outcomes: Electroencephalography. *Anesth Analg.* 2020;130(5):1278-1291.

40. Purdon PL, Pavone KJ, Akeju O, et al. The Ageing Brain: Age-dependent changes in the electroencephalogram during propofol and sevoflurane general anaesthesia. *Br J Anaesth.* 2015;115 Suppl 1:i46-i57.

41. Ben-Israel N, Kliger M, Zuckerman G, Katz Y, Edry R. Monitoring the nociception level: a multi-parameter approach. *J Clin Monit Comput.* 2013;27(6):659-668.

42. Edry R, Recea V, Dikust Y, Sessler DI. Preliminary Intraoperative Validation of the Nociception Level Index: A Noninvasive Nociception Monitor. *Anesthesiology.* 2016;125(1):193-203.

43. Gruenewald M, Ilies C. Monitoring the nociception-anti-nociception balance. *Best Pract Res Clin Anaesthesiol.* 2013;27(2):235-247.

44. Renaud-Roy E, Stockle PA, Maximos S, et al. Correlation between incremental remifentanil doses and the Nociception Level (NOL) index response after intraoperative noxious stimuli. *Can J Anaesth.* 2019;66(9):1049-1061.

45. Stockle PA, Julien M, Issa R, et al. Validation of the PMD100 and its NOL Index to detect nociception at different infusion regimen of remifentanil in patients under general anesthesia. *Minerva Anestesiol.* 2018;84(10):1160-1168.

46. Nasreddine ZS, Phillips NA, Bedirian V, et al. The Montreal Cognitive Assessment, MoCA: a brief screening tool for mild cognitive impairment. *J Am Geriatr Soc.* 2005;53(4):695-699.

47. Zietemann V, Kopczak A, Muller C, Wollenweber FA, Dichgans M. Validation of the Telephone Interview of Cognitive Status and Telephone Montreal Cognitive Assessment Against Detailed Cognitive Testing and Clinical Diagnosis of Mild Cognitive Impairment After Stroke. *Stroke.* 2017;48(11):2952-2957.

48. Bunker L, Hshieh TT, Wong B, et al. The SAGES telephone neuropsychological battery: correlation with in-person measures. *Int J Geriatr Psychiatry.* 2017;32(9):991-999.

49. Marceaux JC, Prosje MA, McClure LA, et al. Verbal fluency in a national sample: Telephone administration methods. *Int J Geriatr Psychiatry.* 2019;34(4):578-587.

50. St-Hilaire A, Hudon C, Vallet GT, et al. Normative data for phonemic and semantic verbal fluency test in the adult French-Quebec population and validation study in Alzheimer's disease and depression. *Clin Neuropsychol.* 2016;30(7):1126-1150.

51. Tombaugh TN, Kozak J, Rees L. Normative data stratified by age and education for two measures of verbal fluency: FAS and animal naming. *Arch Clin Neuropsychol.* 1999;14(2):167-177.

52. Kroenke K, Spitzer RL, Williams JB. The PHQ-9: validity of a brief depression severity measure. *J Gen Intern Med.* 2001;16(9):606-613.

53. Rockwood K, Song X, MacKnight C, et al. A global clinical measure of fitness and frailty in elderly people. *CMAJ.* 2005;173(5):489-495.

54. Abraham P, Courvoisier DS, Annweiler C, et al. Validation of the clinical frailty score (CFS) in French language. *BMC Geriatr.* 2019;19(1):322.

55. McIsaac DI, MacDonald DB, Aucoin SD. Frailty for Perioperative Clinicians: A Narrative Review. *Anesth Analg.* 2020;130(6):1450-1460.

56. Inouye SK, van Dyck CH, Alessi CA, Balkin S, Siegal AP, Horwitz RI. Clarifying confusion: the confusion assessment method. A new method for detection of delirium. *Ann Intern Med.* 1990;113(12):941-948.

57. Inouye SK, Kosar CM, Tommet D, et al. The CAM-S: development and validation of a new scoring system for delirium severity in 2 cohorts. *Ann Intern Med.* 2014;160(8):526-533.

58. Stark PA, Myles PS, Burke JA. Development and psychometric evaluation of a postoperative quality of recovery score: the QoR-15. *Anesthesiology.* 2013;118(6):1332-1340.

59. Rockwood K, Theou O. Using the Clinical Frailty Scale in Allocating Scarce Health Care Resources. *Can Geriatr J.* 2020;23(3):210-215.

60. Manea L, Gilbody S, McMillan D. A diagnostic meta-analysis of the Patient Health Questionnaire-9 (PHQ-9) algorithm scoring method as a screen for depression. *Gen Hosp Psychiatry.* 2015;37(1):67-75.

61. Hou R, Wang H, Chen L, Qiu Y, Li S. POCD in patients receiving total knee replacement under deep vs light anesthesia: A randomized controlled trial. *Brain Behav.* 2018;8(2):e00910.

62. Nagin DS, Odgers CL. Group-Based Trajectory Modeling (Nearly) Two Decades Later. *J Quant Criminol.* 2010;26(4):445-453.

63. Nagin DS, Odgers CL. Group-based trajectory modeling in clinical research. *Annu Rev Clin Psychol.* 2010;6:109-138.
