# Supplementary material for: Effect of electroencephalography-guided anesthesia on neurocognitive disorders in elderly patients undergoing major non-cardiac surgery: A trial protocol The POEGEA trial (POncd Elderly GEneral Anesthesia)
Source: PLoS One. 2021 Aug 10;16(8):e0255852. doi: 10.1371/journal.pone.0255852 (PMC8354438; doi:10.1371/journal.pone.0255852)
Supplement: S3 File — (PDF) [file pone.0255852.s003.pdf]

Centre intégré
universitaire de santé
et de services sociaux
de l'Est-de-
l'Île-de-Montréal
Québec

Le 20 avril 2021

Docteur Philippe Richebé
CIUSSS de l'Est-de-l'Île-de-Montréal
Installation Hôpital Maisonneuve-Rosemont

Objet : Approbation finale du Comité d'éthique de la recherche

Projet n° 2021-2611

Titre : *Effet de l'anesthésie guidée par électroencéphalographie sur les troubles neurocognitifs chez les patients âgés bénéficiant d'une chirurgie non cardiaque majeure: un essai clinique randomisé. POEGEA study (POcd Elderly GEneral Anesthesia)*

Docteur Richebé,

Le Comité d'éthique de la recherche du CIUSSS de l'Est-de-l'Île-de-Montréal a évalué, en comité accéléré, les aspects éthiques de votre projet de recherche. Lors de cette évaluation, les documents suivants ont été examinés, ou le CÉR en a pris acte :

- Formulaire d'information et de consentement (FIC version 05-03-2021.docx) [date : 05 mars 2021]
- Questionnaire (CAM.docx)
- Protocole de recherche (V5_EEG_POCD_protocol 20210308 PR.docx) [date : 08 mars 2021, version : version 5.0]
- Questionnaire (MoCA téléphonique.docx)
- Questionnaire (MoCA.docx)
- Questionnaire (PHQ9_French_for_France.pdf)
- Questionnaire (Qualité de Récupération 15.docx)
- Questionnaire (échelle de fragilité (CFS).docx)
- Questionnaire (fluidité verbale.docx)
- Engagement (Engagnement du chercheur.pdf)

Suite à cette réunion, une approbation conditionnelle vous a été émise en date du 2 avril 2021. Vous nous avez soumis en date du 7 avril 2021, les documents suivants :

- Protocole de recherche (V7_Final_EEG_POCD_protocol 20210406 pour CER (1).docx) [date : 05 avril 2021, version : 7]
- Formulaire d'information et de consentement (FIC version 06-04-2021_CORR_CER (1).docx) [date : 06 avril 2021]
- Autre document (Lettre réponse CER POEGEA 2021-2611 20210406 (1).pdf) [date : 06 avril 2021]

CONCLUSION

Vos réponses et les modifications apportées à votre projet de recherche ont fait l'objet d'une évaluation. Le tout étant jugé satisfaisant, j'ai le plaisir de vous informer que votre projet de recherche a été approuvé à l'unanimité par le Comité d'éthique de la recherche du CIUSSS de l'Est-de-l'Île-de-Montréal.

De plus, le Comité s'est assuré que le projet a fait l'objet d'un examen scientifique dont le résultat est positif et qui a été effectué par une personne ou un comité ayant l'expertise scientifique nécessaire, si applicable.

Les documents que le Comité a approuvés et que vous pouvez utiliser pour la réalisation de votre projet sont les suivants :

- Protocole de recherche (V7_Final_EEG_POCD_protocol 20210406 pour CER (1).docx) [date : 05 avril 2021, version : 7]
- Formulaire d'information et de consentement (FIC version 06-04-2021_CORR_CER (1).docx) [date : 06 avril 2021]
- Questionnaire (CAM.docx)
- Questionnaire (MoCA téléphonique.docx)
- Questionnaire (MoCA.docx)
- Questionnaire (PHQ9_French_for_France.pdf)
- Questionnaire (Qualité de Récupération 15.docx)
- Questionnaire (échelle de fragilité (CFS).docx)
- Questionnaire (fluidité verbale.docx)

PRÉCISIONS

- Le mot "survnue" pourra être corrigé dans la version à faire signer par les participants.

Cette approbation éthique est valide pour un an à compter de la date de la présente lettre, date de l'approbation finale. Deux mois avant la date d'échéance, vous devrez faire une demande de renouvellement auprès du Comité, en utilisant le document du Comité prévu à cet effet.

Il est à noter qu'aucun membre du comité d'éthique participant à l'évaluation et à l'approbation de ce projet n'est impliqué dans celui-ci.

Dans le cadre du suivi continu, le Comité vous demande de vous conformer aux exigences suivantes en utilisant les formulaires du Comité prévus à cet effet et de soumettre, le cas échéant :

1. toute demande de modification au projet de recherche ou à tout document approuvé par le Comité pour la réalisation de votre projet;
2. tout nouveau renseignement ou toute modification à l'équilibre clinique susceptible d'affecter l'intégrité ou l'éthicité du projet de recherche, d'accroître les risques et les inconvénients pour les participants, de nuire au bon déroulement du projet ou d'avoir une incidence sur le désir d'un participant de continuer à participer au projet;
3. les réactions indésirables qui rencontrent tous les critères suivants :
   a) Cette réaction a eu lieu dans notre établissement ou dans un établissement pour lequel le CÉR a juridiction; b) Il doit s'agir de réaction indésirable ou de réaction indésirable grave ou de réaction indésirable et inattendue; c) Cette réaction doit être reliée ou possiblement, probablement ou certainement reliée au médicament à l'étude ou à une procédure de l'étude.
4. tout accident survenu dans votre site;
5. l'interruption prématurée du projet de recherche, qu'elle soit temporaire ou permanente;
6. tout problème constaté à la suite d'une activité de surveillance ou de vérification menée par un tiers et susceptible de remettre en question l'intégrité ou l'éthicité du projet de recherche;
7. toute suspension ou annulation de l'approbation octroyée par un organisme de subvention ou de réglementation;
8. toute procédure en cours de traitement d'une plainte ou d'une allégation de manquement à l'intégrité ou à l'éthicité ainsi que des résultats de la procédure;
9. toute déviation au projet de recherche susceptible d'augmenter le niveau de risque ou susceptible d'influer sur le bien-être du participant ou d'entacher le consentement du participant;
10. une demande de renouvellement annuel de l'approbation du projet de recherche;
11. le rapport de la fin du projet de recherche.

Nous vous rappelons que la présente décision vaut pour une année et peut être suspendue ou révoquée en cas de non-respect de ces exigences.

Le CÉR achemine votre projet au bureau de la personne formellement mandatée responsable pour obtenir l'autorisation de la réalisation de votre projet au CIUSSS de l'Est-de-l'Île-de-Montréal. Il est entendu que vous ne pouvez commencer la réalisation de votre projet avant d'avoir obtenu cette autorisation.

De plus, nous vous rappelons que vous devez conserver pour une période d'au moins un an suivant la fin du projet, un répertoire distinct comprenant les noms, prénoms, coordonnées, date du début et de fin de la participation de chaque sujet de recherche.

Le Comité d'éthique de la recherche du CIUSSS de l'Est-de-l'Île-de-Montréal est désigné par le ministre de la Santé et des Services sociaux (MSSS) pour les fins d'application de l'article 21 du Code civil du Québec. Il adhère aux directives publiées dans l'Énoncé de politique des trois conseils (ÉPTC 2, 2014), au Plan d'action ministériel en éthique de la recherche et en intégrité scientifique (MSSS 1998) et aux exigences édictées pour les comités d'éthique de la recherche à la Partie C, Titre 5 du Règlement sur les aliments et

drogues du Canada (C.R.C. ch.870), ou à la définition dans l' *Arrêté d'urgence sur les essais cliniques d'instruments médicaux et de drogues en lien avec la COVID-19*. Il agit également en conformité avec les standards du *United States Code of Federal Regulations* encadrant la recherche avec des participants humains. Le Comité fonctionne de manière compatible avec les standards internationaux en appliquant, notamment, la Ligne directrice de l'ICH adoptée par Santé Canada : Les bonnes pratiques cliniques.

De plus, nous vous avisons que les règles de fonctionnement du Comité d'éthique de la recherche du CIUSS de l'Est-de-l'Ile-de-Montréal satisfont aux exigences de Santé Canada relatives à l'Attestation du comité d'éthique pour la recherche. La recherche nécessitant un numéro de « US Federal Wide Assurance » est effectuée au CIUSSS de l'Est-de-l'Ile-de-Montréal sous les numéros suivants : FWA00001935 et IRB00002087.

Avec l'expression de nos sentiments les meilleurs.

Vanessa Chenel, Ph. D.
Cadre conseil en éthique
Présidente du Comité d'éthique de la recherche
CIUSSS de l'Est-de-l'Île-de-Montréal
5415, boul. de l'Assomption
Tél. : 514-252-3400, poste 5708
cer.cemtl@ssss.gouv.qc.ca
